# Allylic Hydrogen Acidity of 1-Butene Derivatives Coordinated to Transition Metals—A Mechanistic Insight Including Carbonyl–Olefin Metathesis

**DOI:** 10.1021/acs.inorgchem.4c05297

**Published:** 2025-02-25

**Authors:** Kaveh Farshadfar, Zonghang Song, Kari Laasonen

**Affiliations:** Department of Chemistry and Material Science, School of Chemical Engineering, Aalto University, 02150 Espoo, Finland

## Abstract

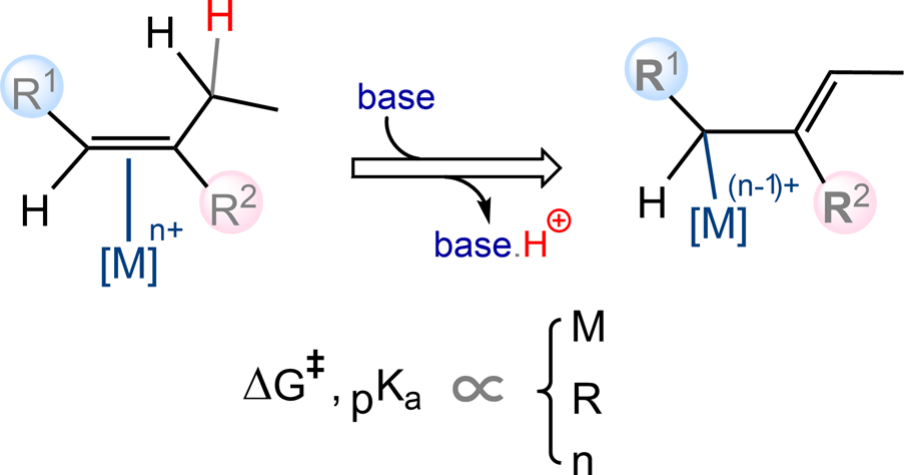

The coordination of organic molecules to transition metals significantly alters the electron density distribution, influencing the acidity of specific hydrogen atoms. This study scrutinizes the acidity of allylic hydrogens in transition metal-coordinated alkenes, delving into the factors that govern allylic proton abstraction. Employing density functional theory, we investigate the effects of various parameters, including the electronic nature of substituents on the vinylic carbons of the alkene, the oxidation state of the metal, and the identity of the transition metal center on the allylic hydrogens’ acidity. Our findings reveal that the impact on the acidity of allylic hydrogens in alkenes coordinated to gold(III), a third-row transition metal, is considerably substantial both kinetically and thermodynamically. Conversely, the impact is minimal for cobalt(III) from the first row and moderate for rhodium(III) from the second row of transition metals. Furthermore, our results indicate that electron-withdrawing substituents on vinylic carbons generally enhance the acidity of allylic hydrogens. The influence of oxidation state is also profound, as gold(I) exhibits markedly weaker effects compared to gold(III). To illustrate the practical application of these insights, we present a case study involving the use of AuCl_3_ to catalyze an organic transformation [*Chem. Eur. J.* 2020, 26, 1941–1946], elucidating the mechanism initiated by the deprotonation of the allylic hydrogen in the coordinated alkene.

## Abstract 2

This study scrutinizes the acidity of allylic hydrogens in transition metal-coordinated alkenes, delving into the factors that govern allylic proton abstraction. We present a case study involving the use of AuCl_3_ to catalyze an organic transformation, elucidating the mechanism initiated by the deprotonation of the allylic hydrogen in the coordinated alkene.

## Introduction

Coordination of an organic molecule to a transition metal can profoundly alter the electron density distribution in specific regions of the molecule, thereby influencing the acidity of certain hydrogen atoms. This influence of transition metals offers significant potential for further research.

**Scheme 1:**
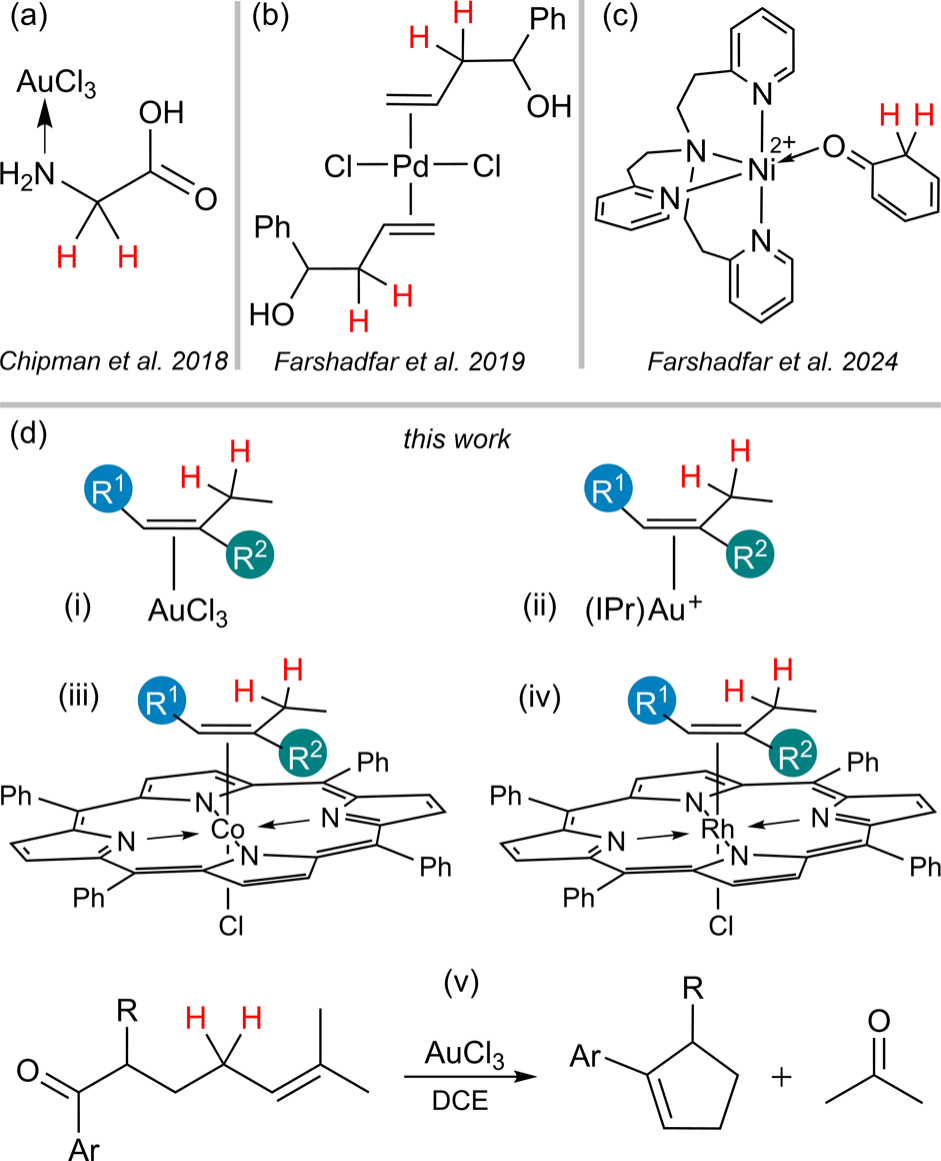
Mechanistic Investigations into the Enhanced Acidity of α- and Allylic Hydrogens upon Coordination to Transition Metals

In a 2018 study on the reduction of gold(III) by the amino acid glycine,^1^ we demonstrated that coordination of the amino acid to the gold(III) center via its nitrogen atom results in the C^α^ hydrogen becoming significantly more acidic than the hydrogen of its carboxylic group, to the extent that even a weak base, such as bulk water, can deprotonate it (Scheme 1a). The study revealed that amines can undergo deprotonation via this mechanism as well. In another study in 2019,^2^ we extended our findings to encompass allylic hydrogens in alkenes, revealing their considerable acidity upon coordination to Pd(II). This approach was employed to propose a mechanism for alkene isomerization catalyzed by PdCl_2_ (Scheme 1b). Furthermore, the study demonstrated that AuCl_3_ can catalyze double-bond migration, showing higher acidity for the allylic hydrogens within coordinated alkenes. More recently, in 2024,^3^ as part of the mechanistic study on nickel(II)-catalyzed benzene hydroxylation with H_2_O_2_, we reported the substantial acidity of the C^α^ hydrogen in benzene oxide upon coordination to the nickel(II) center via the oxygen atom (Scheme 1c).

Building upon our previous study that highlighted the notable acidity of allylic hydrogens in certain transition metal-coordinated alkenes, we delve into the parameters influencing the ease of metal-coordinated allylic proton abstraction. The identity of the transition metal, its oxidation state, the electronic nature of the substituents on the vinylic carbons (α and β-carbons), and the nature of the supporting ligand^4^ are expected to influence allylic hydrogen acidity. To comprehensively elucidate these intricate relationships, we undertook comprehensive DFT studies at the SMD/M06/def2-TZVP//SMD/M06/SDD,6-31G(d) level of theory. Accordingly, nine diverse substituents, covering a broad spectrum from electron-withdrawing to electron-donating groups, were introduced on the vinylic carbons of 1-butene as an olefin. The activation energy barrier for allylic proton abstraction by bulk water in the AuCl_3_-coordinated alkene, as well as the thermodynamic stability of the resulting anion, were then calculated. Subsequently, similar calculations were performed for Au(I)IPr (Scheme 1d^ii^), meso-tetraphenylporphyrin-Co(III) chloride (Scheme 1d^iii^), and meso-tetraphenylporphyrin-Rh(III) chloride (Scheme 1d^iv^) complexes with selected substituents. Finally, we showcase the utility of this approach by presenting a compelling example that unravels the mechanistic intricacies of a carbonyl–olefin metathesis transformation catalyzed by AuCl_3_ (Scheme 1d^v^).

## Results and Discussion

**Scheme 2:**
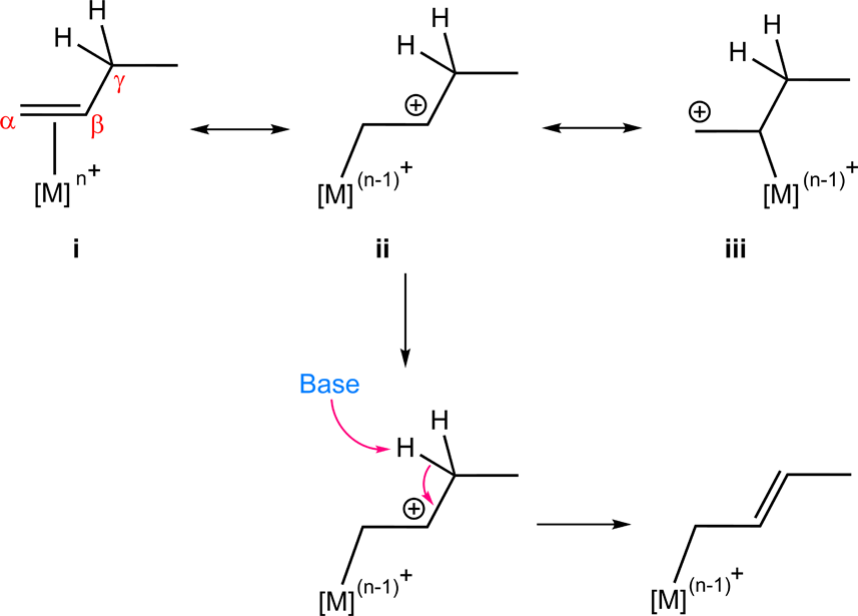
Resonance Structures of Alkene π-Complexes and Mechanism of Resonance Structure **ii** Contributing to the Acidity of Allylic Hydrogens

When an olefin coordinates to a transition metal (structure **i** in Scheme 2), the allylic hydrogens (hydrogens attached to the γ-carbon) may become acidic due to the contribution of resonance structure **ii**, wherein the metal establishes a σ-bond with the α-carbon atom, and the positive charge is localized on the β-carbon. Therefore, upon losing a proton from the γ-carbon, the bonding electrons participate in the π-bond with the positively charged carbon (β-carbon), leading to the formation of the C=C double bond. As a result, π-complexes with a high positive charge on the β-carbon are expected to undergo facile deprotonation. Electron-donating substituents on the vinylic carbons stabilize the π-complex by enhancing electron donation to the metal center. Nonetheless, the delocalization of the β-carbon’s positive charge can suppress its acidity. Conversely, electron-withdrawing substituents on the vinylic carbons can destabilize the π-complex, while amplifying a greater positive charge on the β-carbon. Therefore, it is worthwhile to investigate the impact of various substituents on the ease of deprotonation of the allylic hydrogen in transition metal-coordinated alkenes.

### Allylic Hydrogen Acidity in 1-Butene Derivatives Coordinated to AuCl_3_

**Scheme 3:**
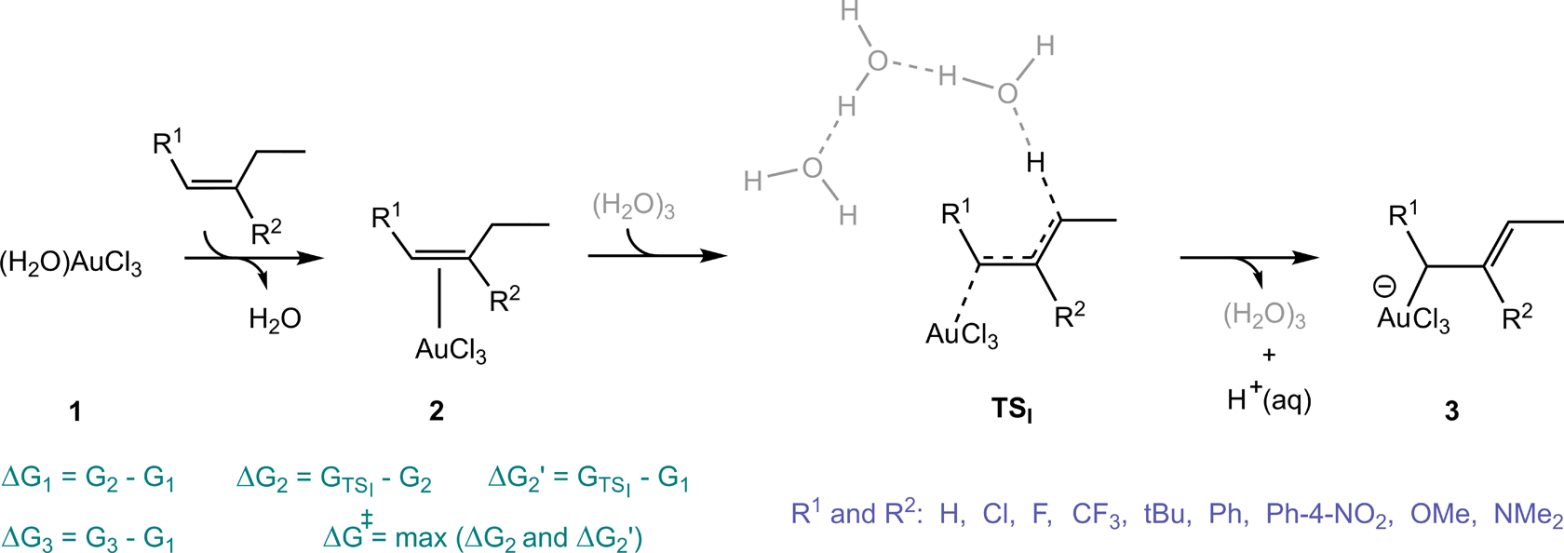
Investigated Mechanism and the Approach for Calculating Free Energies for the Deprotonation of Allylic Hydrogen in 1-Butene Derivatives Coordinated to AuCl_3_

In pursuit of understanding how substituent effects influence the acidity of allylic hydrogens, a diverse array of electron-withdrawing and electron-donating substituents including H, Cl, F, CF_3_, ^t^Bu, Ph, Ph-4-NO_2_, OMe, and NMe_2_ were introduced onto the vinylic carbons of 1-butene, with AuCl_3_ utilized as the catalyst (Scheme 3). The relative free energies of the resulting π-complexes (**2**), compared to (H_2_O)AuCl_3_ (**1**), were computed initially to evaluate their stability. Given that water was selected as the solvent and that a cluster of three water molecules [(H_2_O)_3_] is a well-accepted model for bulk water,^3,5−11^ we employed it as the base for deprotonation to identify the transition state structures. For all samples, the corresponding transition structures for deprotonation (**TS**_**I**_) were located. Experimental data on the free energy of protonation in water was employed to determine the relative thermodynamic stability of complex **3**. Table 1 presents the computational results corresponding to Scheme 3. In cases where R^1^ = NMe_2_ and R^2^ = ^t^Bu, OMe, or NMe_2_, gold(III) underwent reduction upon reaction with the alkene prior to deprotonation, and for R^1^ = NMe_2_ and R^2^ = Cl, after deprotonation; therefore, these data points were excluded from the analysis.

**Table 1:** Calculated Thermodynamic and Kinetic Values for Various R^1^ and R^2^ Substituents on 1-Butene Coordinated to Au^III^, Based on the Proposed Approach Outlined in Scheme 3^a^

R^1^	R^2^	Δ*G*_1_	Δ*G*_2_	Δ*G*_3_	Δ*G*^‡^	p*K*_a_
H	H	–12.2	15.4	–27.7	15.4	–11.4
H	Cl	–8.4	12.3	–27.0	12.3	–13.6
H	F	–12.1	13.1	–26.0	13.1	–10.2
H	CF_3_	4.2	11.8	–21.3	16.0	–15.6
H	^t^Bu	–8.2	12.7	–19.7	12.7	–8.4
H	Ph	–8.0	11.4	–23.5	11.4	–11.4
H	Ph-4-NO_2_	–4.3	11.5	–23.2	11.5	–13.9
H	OMe	–19.0	22.6	–20.0	22.6	–0.7
H	NMe_2_	–36.7	31.5	–17.1	31.5	14.4
Cl	H	–4.1	14.3	–20.2	14.3	–11.8
Cl	Cl	2.5	9.2	–22.4	11.7	–16.4
Cl	F	–0.9	9.9	–21.5	9.9	–15.1
Cl	CF_3_	10.3	8.7	–20.7	19.0	–15.2
Cl	^t^Bu	–0.4	11.5	–17.4	11.5	–12.5
Cl	Ph	0.0	9.9	–20.1	9.9	–14.7
Cl	Ph-4-NO_2_	3.3	10.2	–20.2	13.5	–14.8
Cl	OMe	–10.9	17.7	–18.3	17.7	–5.4
Cl	NMe_2_	–25.3	26.6	–15.3	26.6	7.3
F	H	–6.6	15.6	–23.9	15.6	–12.7
F	Cl	0.8	8.9	–25.3	9.7	–18.5
F	F	–5.0	10.3	–26.0	10.3	–15.4
F	CF_3_	10.9	11.1	–18.6	22.0	–13.6
F	^t^Bu	–1.6	10.2	–19.5	10.2	–13.1
F	Ph	–1.6	10.3	–22.0	10.3	–15.0
F	Ph-4-NO_2_	1.2	11.5	–21.3	11.5	–15.6
F	OMe	–14.0	17.1	–21.2	17.1	–5.2
F	NMe_2_	–37.0	30.4	–22.9	30.4	10.3
CF_3_	H	–1.1	10.8	–21.2	10.8	–14.7
CF_3_	Cl	5.7	8.3	–22.9	14.0	–16.8
CF_3_	F	0.9	8.9	–21.1	9.8	–15.5
CF_3_	CF_3_	13.7	6.9	–19.1	20.6	–14.0
CF_3_	^t^Bu	1.1	10.9	–15.0	12.0	–11.0
CF_3_	Ph	2.3	9.5	–18.6	11.8	–13.6
CF_3_	Ph-4-NO_2_	5.8	9.7	–20.2	15.5	–14.8
CF_3_	OMe	–11.9	22.0	–14.9	22.0	–2.2
CF_3_	NMe_2_	–21.1	30.2	–9.5	30.2	8.5
^t^Bu	H	–10.0	18.8	–20.8	18.8	–7.9
^t^Bu	Cl	0.2	13.4	–18.3	13.6	–13.4
^t^Bu	F	–4.2	13.3	–18.5	13.3	–10.5
^t^Bu	CF_3_	6.4	12.5	–15.9	18.9	–11.7
^t^Bu	^t^Bu	–4.9	14.4	–15.4	14.4	–7.7
^t^Bu	Ph	–5.0	12.7	–20.7	12.7	–11.5
^t^Bu	Ph-4-NO_2_	–3.5	13.8	–19.2	13.8	–11.5
^t^Bu	OMe	–17.7	24.0	–17.5	24.0	0.1
^t^Bu	NMe_2_	–31.3	32.7	–15.5	32.7	11.6
Ph	H	–9.4	18.8	–20.4	18.8	–8.1
Ph	Cl	–5.8	13.9	–24.0	13.9	–13.3
Ph	F	–4.4	13.8	–18.0	13.8	–10.0
Ph	CF_3_	6.9	15.0	–14.7	21.9	–10.8
Ph	^t^Bu	–1.7	7.2	–17.7	7.2	–11.7
Ph	Ph	–4.0	14.1	–18.0	14.1	–10.3
Ph	Ph-4-NO_2_	–2.7	11.2	–18.5	11.2	–11.6
Ph	OMe	–13.2	22.8	–15.1	22.8	–1.4
Ph	NMe_2_	–30.6	29.0	–13.3	29.0	12.7
Ph-4-NO_2_	H	–6.3	18.0	–19.7	18.0	–9.8
Ph-4-NO_2_	Cl	2.4	12.8	–18.2	15.2	–13.3
Ph-4-NO_2_	F	–3.0	14.3	–17.9	14.3	–10.9
Ph-4-NO_2_	CF_3_	7.8	13.8	–16.9	21.6	–12.4
Ph-4-NO_2_	^t^Bu	–1.6	7.6	–17.5	7.6	–11.7
Ph-4-NO_2_	Ph	–1.9	12.3	–17.2	12.3	–11.2
Ph-4-NO_2_	Ph-4-NO_2_	–1.6	12.9	–18.5	12.9	–12.4
Ph-4-NO_2_	OMe	–9.7	14.3	–14.3	14.3	–3.4
Ph-4-NO_2_	NMe_2_	–26.4	28.7	–9.2	28.7	12.6
OMe	H	–18.1	22.2	–27.9	22.2	–7.2
OMe	Cl	–6.2	13.8	–26.8	13.8	–15.1
OMe	F	–13.3	14.1	–29.5	14.1	–11.9
OMe	CF_3_	–1.4	18.4	–22.0	18.4	–15.1
OMe	^t^Bu	–17.0	22.7	–25.0	22.7	–5.9
OMe	Ph	–9.4	14.8	–25.3	14.8	–11.7
OMe	Ph-4-NO_2_	–4.7	15.7	–22.2	15.7	–12.8
OMe	OMe	–20.7	19.3	–24.8	19.3	–3.0
OMe	NMe_2_	–38.5	30.8	–24.1	30.8	10.6
NMe_2_	H	–33.7	28.3	–42.3	28.3	–6.3
NMe_2_	F	–27.2	26.3	–38.1	26.3	–8.0
NMe_2_	CF_3_	–16.6	30.2	–31.8	30.2	–11.1
NMe_2_	Ph	–22.0	21.5	–45.5	21.5	–17.2
NMe_2_	Ph-4-NO_2_	–20.2	23.1	–38.9	23.1	–13.7

a Free energies are given in kcal/mol, and the p*K*_a_ values are for the allylic hydrogen.

**Figure 1:**
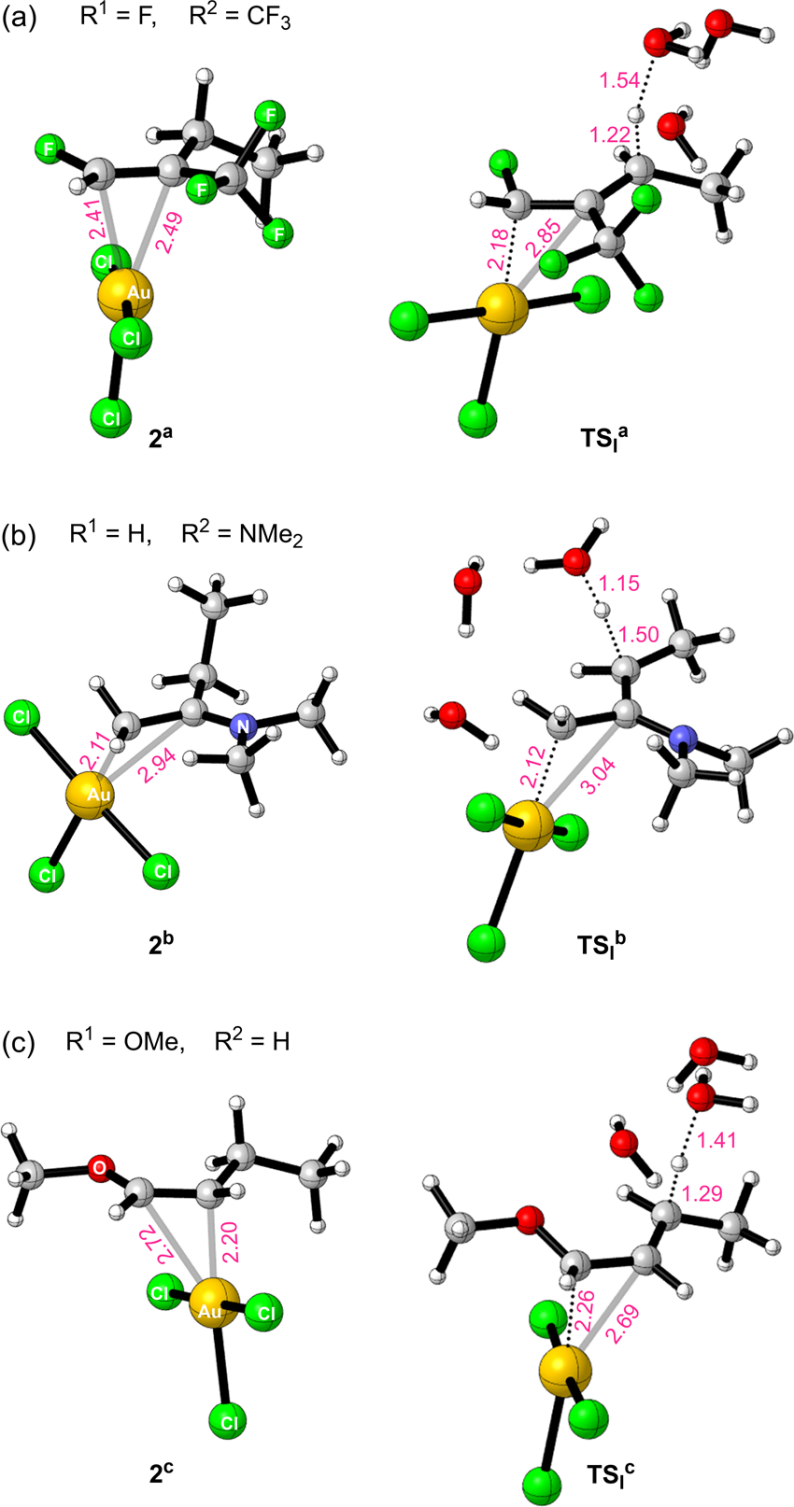
3D Representations of selected examples of species **2** and their corresponding transition state structures for proton capture by bulk water (**TS**_**I**_). The selected distances (Å) are annotated in pink.

The formation of the π-complex in the studied samples exhibits a wide range of thermodynamic stability, varying from highly exothermic at −38.5 kcal/mol to endothermic at 13.7 kcal/mol. Electron-donating substituents, particularly strong π-donors such as NMe_2_ and OMe, whether at the R^1^ or R^2^ positions, enhance the thermodynamic stability of the metal complex **2** by delocalizing the induced positive charge. A more electron-donating R^1^ increases the weight of resonance contributor **iii**, while a more electron-donating R^2^ favors resonance contributor **ii**. Conversely, electron-withdrawing R^1^ and R^2^ substituents destabilize species **2** and amplify the contribution of resonance structure **i**. Some examples illustrating the dominance of each type of these resonance contributors are provided in Figure 1.

**Figure 2:**
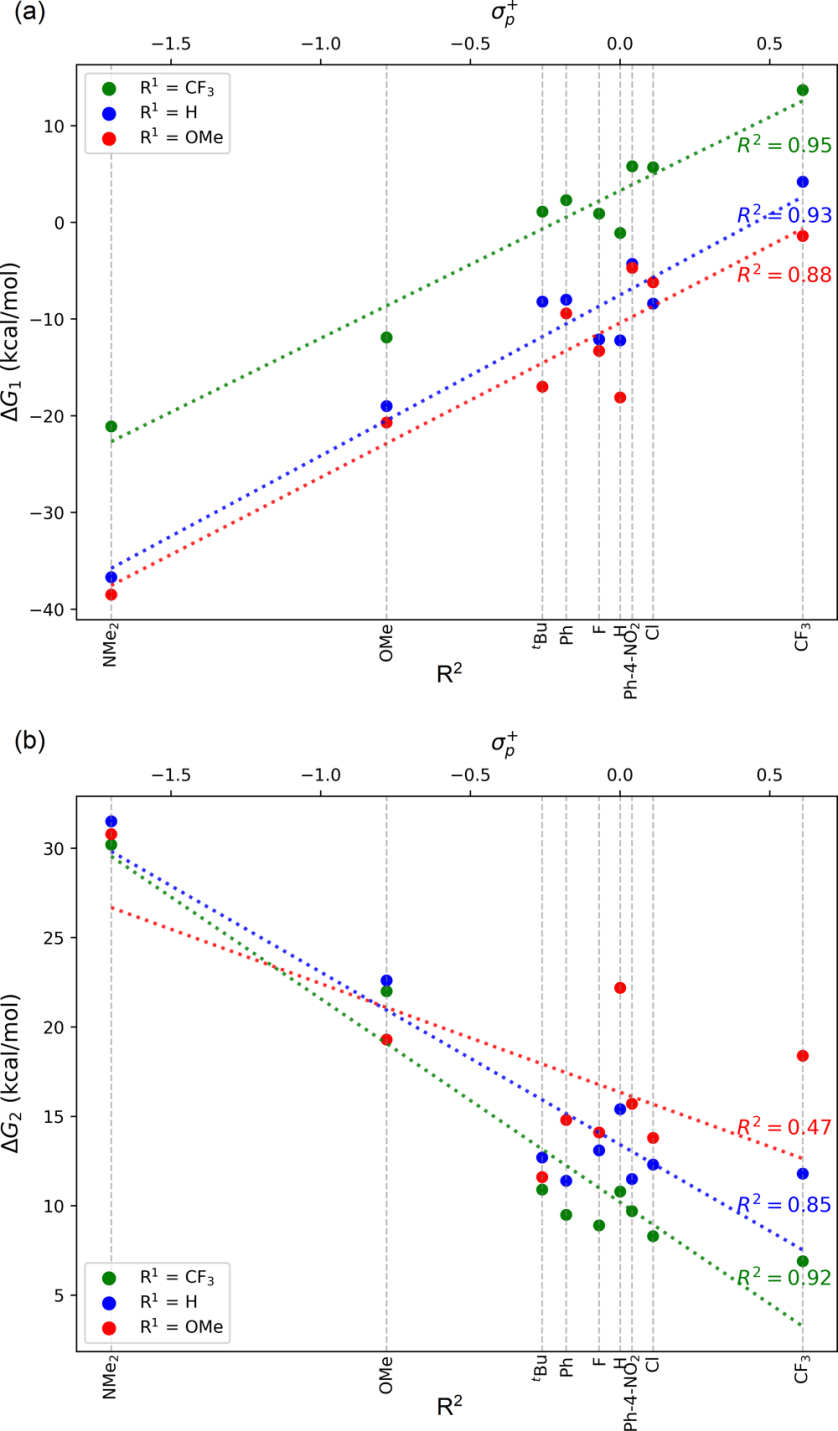
(a) Correlation between the relative free energy of the π-complex (Δ*G*_1_) and the Hammett σ_*p*_^+^ values of the R^2^ substituents, with R^1^ = H, CF_3_, or OMe. (b) Correlation between Δ*G*_2_ and the Hammett σ_*p*_^+^ values of the R^2^ substituents, with R^1^ = H, CF_3_, or OMe.

Figure 2 illustrates the relationship between the electronic properties of the substituents and both the stability of the π-complex and the free energy difference between the π-complex and the transition state for deprotonation. For this purpose, samples with R^1^ = CF_3_ as a strong electron-withdrawing group, R^1^ = H serving as a reference, and R^1^ = OMe as a strong electron-donating group were selected. (Due to incomplete data for R^1^ = NMe_2_, this substituent was excluded as a representative electron-donating group.)

Figure 2a depicts the correlation between the relative free energy of the π-complex (Δ*G*_1_) and the Hammett σ_*p*_^+^ values^12^ of the R^2^ substituents, with R^1^ held constant. σ_*p*_^+^ parameter was employed as a quantitative indicator of the electron-withdrawing/donating effects of the substituents. σ_*p*_^+^ parameter was chosen because the resonance structures **ii** and **iii**, where the alkene π-bond is polarized with a positive charge on the carbon atom, are the predominant structures in the studied data set. A significant linear relationship is observed between Δ*G*_1_ and σ_*p*_^+^ values, underscoring the substantial influence of the substituent’s electronic properties on the π-complex stability. Notably, data points where R^2^ = H deviate somewhat from the trend. Structural analysis of the complexes **2** shows that the distance between the Au(III) center and the β-carbon is noticeably shorter in cases where R^2^ is hydrogen (Figure S1). This could be due to the reduced steric effects of hydrogen, ultimately leading to a more stable π-complexes. Excluding these outliers results in a markedly stronger correlation, as demonstrated in Figure S2.

Δ*G*_2_ represents the free energy difference between the transition structure (**TS**_**I**_) and **2**. Figure 2b illustrates the relationship between the Δ*G*_2_ energy and the Hammett σ_*p*_^+^ parameter for samples with R^1^ = H, CF_3_, OMe, and varying R^2^. The plot reveals that substrates with strongly electron-withdrawing R^1^ and R^2^ groups exhibit the smallest energy difference between the π-complex and the transition state. As substituents with stronger electron-donating properties are introduced, this energy gap increases. Overall, no strong linear correlation is observed between Δ*G*_2_ and the  values, particularly when R^2^ is a strong electron donor such as NMe_2_ or OMe. These electron-donating groups transfer electron density to the p_*z*_ orbital of the β-carbon in resonance contributor **ii** in Scheme 2, significantly reducing the acidity of the allylic hydrogen atoms. Thus, the influence of R^1^ becomes negligible during deprotonation in such cases. For samples with R^2^ = H, the previously discussed enhanced stability of their π-complexes and the closer proximity of the β-carbon to the gold center make gold detachment more challenging, as the gold center must shift toward the α-carbon during the transition states. Consequently, substrates with R^2^ = H exhibit higher Δ*G*_2_ values compared to those with σ_*p*_^+^ values in a similar range.

Electron-donating R^1^ and R^2^ substituents produce complex **2** exergonically while elevating the deprotonation activation energy. When R^1^ is a strong electron donor, during the deprotonation transition state, formation of the π-bond between the β and γ-carbons necessitates the gold center moving from the β-carbon toward the α-carbon, resulting in an energetically unfavorable rearrangement. Figure 1, depicting , provides an illustrative example of this type of transition state. Conversely, a strong electron-donating R^2^ delocalizes the positive charge on the β-carbon, thereby reducing the acidity of the allylic hydrogens.

The activation energy for each sample is the energy difference between the transition state for deprotonation and the resting state of the catalyst, which for the majority of samples is species **2**, but for a smaller number, it is the starting materials. Compounds exhibiting lower free energy differences between **TS**_**I**_ and complex **2** (Δ*G*_2_) do not necessarily have lower activation barriers (Δ*G*^‡^), as the formation of **2** might be endergonic for them. One such example is 2-butene decorated with both R^1^ and R^2^ with CF_3_, featuring a π-complex energy of 13.7 kcal/mol. Therefore, it can be concluded that alkenes with Δ*G*_1_ energies near thermoneutral undergo allylic hydrogenation more easily. These alkenes encompass those bearing either a single strong electron-withdrawing group like CF_3_, or two moderately electron-withdrawing groups such as F and Cl.

**Figure 3:**
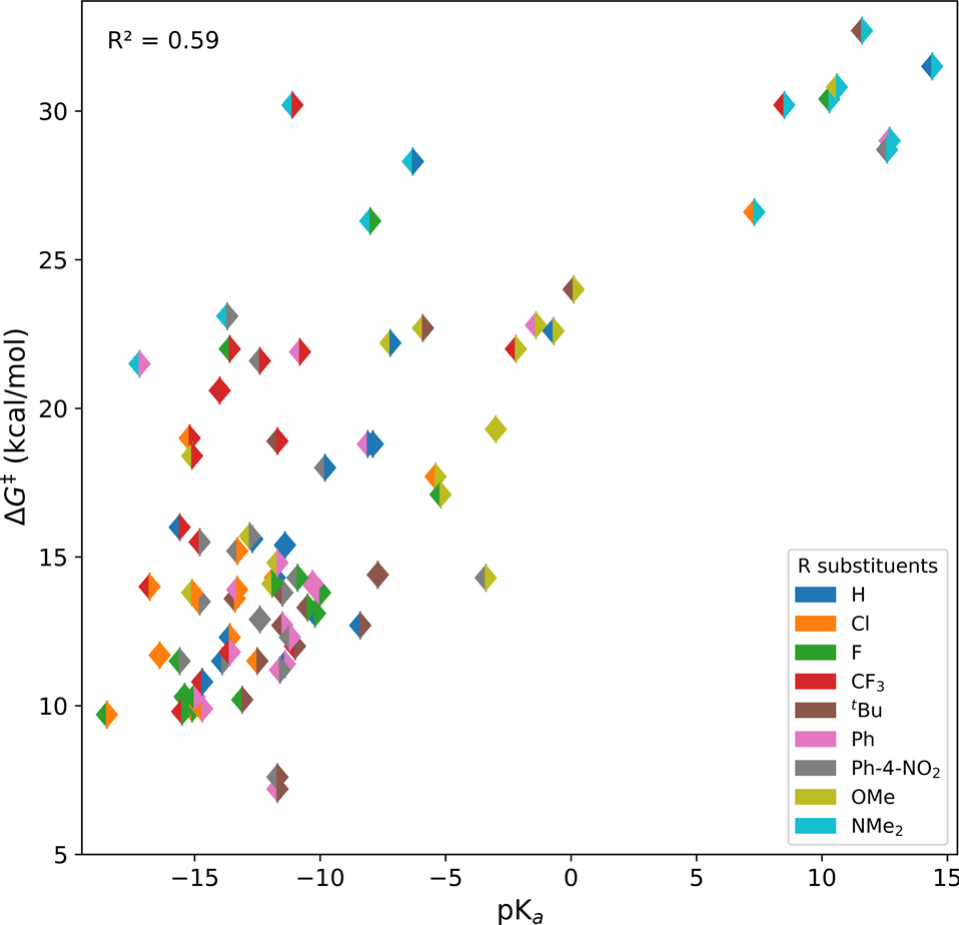
Plot of p*K*_a_ versus Δ*G*^‡^ for the data presented in Table 1. Each sample is represented by a diamond, where the left triangle indicates the R^1^ substituent and the right triangle represents the R^2^ substituent.

In Table 1, we have also included p*K*_a_ values, determined by calculating the free energy difference between complex **3** and the most stable species prior to deprotonation (complex **1** or **2**), while considering the experimental free energy of proton solvation in water. The p*K*_a_ values were then obtained using the equation Δ*G* = −RTln *K*_a_. Remarkably, the p*K*_a_ values for the majority of samples were observed to be significantly negative, underscoring the extreme acidity of the allylic hydrogens in their AuCl_3_-coordinated structures, whereas for free aliphatic alkenes, the p*K*_a_ values have been reported to exceed 40 (in DMSO).^13^ Moreover, these p*K*_a_ values exhibit a linear relationship with Δ*G*^‡^ values, with a correlation coefficient of 0.59, as shown in Figure 3. However, if the samples with R^1^ = NMe_2_ are treated as outliers, removing them increases the correlation coefficient to 0.73 (Figure S3).

### Allylic Hydrogen Acidity in 1-Butene Derivatives Coordinated to Au(IPr)^+^

**Scheme 4:**
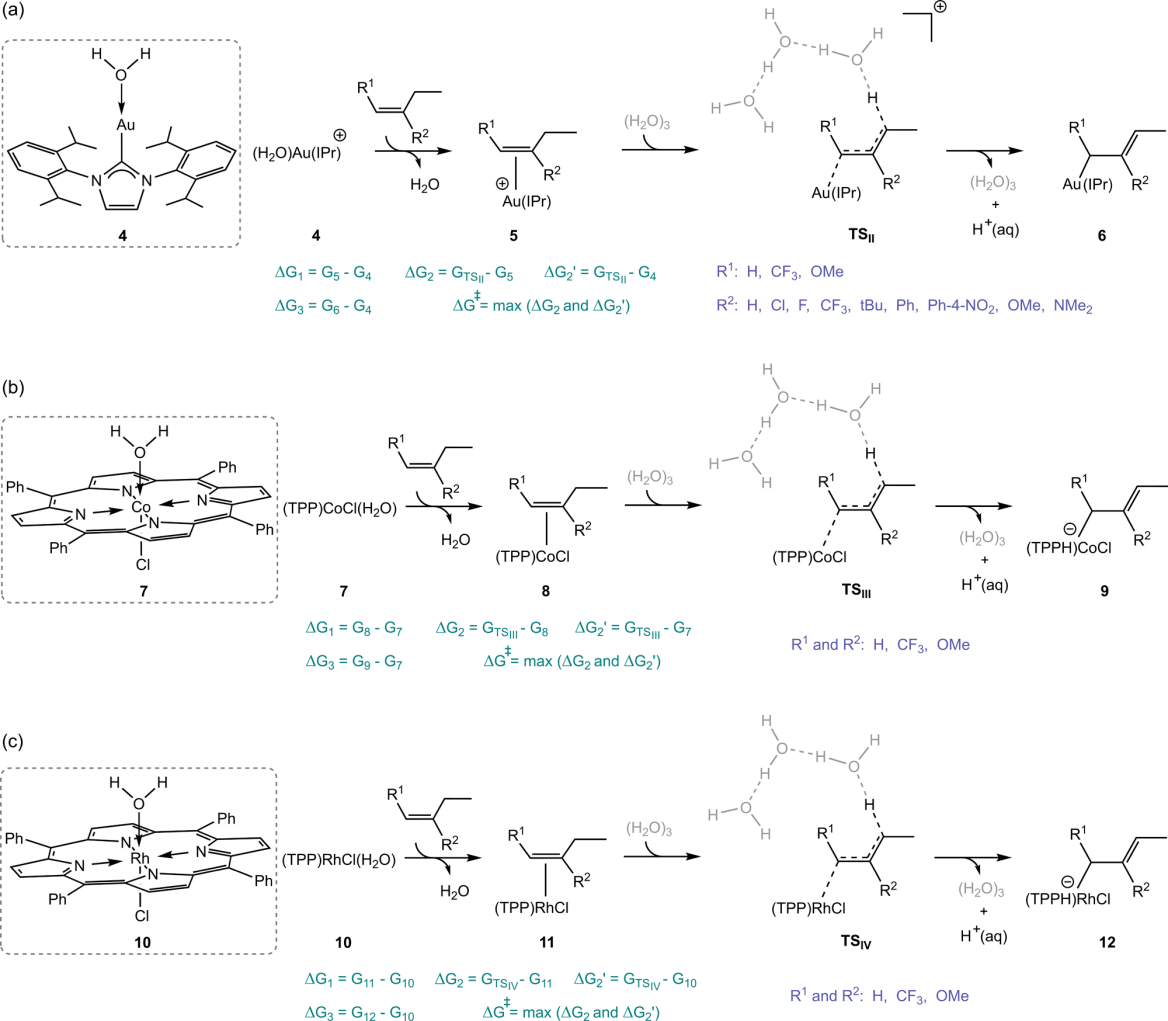
Approach for Calculating Free Energies of Deprotonation of Allylic Hydrogen in 1-Butene Derivatives Coordinated to Au(I), Co(III), and Rh(III) Transition Metals

To elucidate the impact of oxidation state on allylic deprotonation, we expanded our study to Au(I) complexes, employing Au(IPr)^+^ as a catalyst (Scheme 4a). Here, IPr refers to N,*N*′-bis(2,6-diisopropylphenyl)imidazol-2-ylidene, a common ligand for Au(I) complexes.^14−16^ This investigation encompassed a subset of compounds featuring R^1^ = CF_3_, H, and OMe, while maintaining all R^2^ substituents from the previous series. Although a similar trend to that observed with the AuCl_3_ catalyst is noted for Au(IPr)^+^, the deprotonation energy barriers are markedly higher compared to those with AuCl_3_. Additionally, the p*K*_a_ values for 1-butene derivatives coordinated to Au(IPr)^+^ are significantly higher than those coordinated to AuCl_3_, reflecting decreased acidity due to the lower oxidation state. This discrepancy can be attributed to the reduced electron deficiency associated with Au(I) compared to Au(III). Detailed results related to this study are presented in Table 2.

**Table 2:** Calculated Thermodynamic and Kinetic Values for Selected Substituents on 1-Butene Coordinated to Au^I^, Based on the Approach Outlined in Scheme 4a^a^

R^1^	R^2^	Δ*G*_1_	Δ*G*_2_	Δ*G*_3_	Δ*G*^‡^	p*K*_a_
H	H	–9.3	32.8	14.3	32.8	16.3
H	Cl	–4.5	29.7	12.2	29.7	12.2
H	F	–6.0	29.3	13.6	29.3	14.4
H	CF_3_	–2.7	33.0	15.1	33.0	13.0
H	^t^Bu	–7.7	36.0	19.0	36.0	19.6
H	Ph	–8.6	33.9	14.3	33.9	16.8
H	Ph-4-NO_2_	–6.3	31.6	13.8	31.6	14.7
H	OMe	–11.8	35.0	12.8	35.0	18.0
H	NMe_2_	–18.2	42.2	16.3	42.2	25.3
CF_3_	H	–6.7	31.8	7.0	31.8	10.0
CF_3_	Cl	2.8	23.1	6.1	25.8	4.5
CF_3_	F	–1.8	26.3	7.4	26.3	6.7
CF_3_	CF_3_	1.9	29.3	7.9	31.2	5.8
CF_3_	^t^Bu	–3.3	31.6	13.6	31.6	12.4
CF_3_	Ph	–5.0	29.8	7.2	29.8	8.9
CF_3_	Ph-4-NO_2_	–2.6	25.8	6.9	25.8	7.0
CF_3_	OMe	–10.3	33.6	7.6	33.6	13.1
CF_3_	NMe_2_	–13.8	41.0	16.8	41.0	22.4
OMe	H	–9.9	41.6	19.9	41.6	21.8
OMe	Cl	–7.0	37.6	14.8	37.6	16.0
OMe	F	–0.6	29.1	16.0	29.1	12.2
OMe	CF_3_	–6.1	43.6	20.2	43.6	19.3
OMe	^t^Bu	–9.1	42.2	21.2	42.2	22.2
OMe	Ph	–11.6	42.2	19.9	42.2	23.1
OMe	Ph-4-NO_2_	–5.9	37.9	20.9	37.9	19.6
OMe	OMe	–7.7	31.0	17.4	31.0	18.4
OMe	NMe_2_	–16.9	42.3	15.7	42.3	23.9

a Free energies are given in kcal/mol, and the p*K*_a_ values are for the allylic hydrogen.

### Allylic Hydrogen Acidity in 1-Butene Derivatives Coordinated to Cobalt(III) Porphyrin

We subsequently opted for (TPP)CoCl (where TPP = meso-tetraphenylporphyrin dianion) to assess the allylic hydrogen acidity in 1-butene derivatives when coordinated to Co(III), comparing it to that observed with AuCl_3_. The synthesis of this cobalt complex has been previously documented.^17,18^ For this analysis, we limited substitutions at the R^1^ and R^2^ positions to H, OMe as an electron-donating group, and CF_3_ as an electron-withdrawing group. The DFT results, detailed in Table 3, indicate that unlike gold(III), both the activation energy required for allylic hydrogen deprotonation and the p*K*_a_ values are significantly higher in the Co(III) complex. So far, our findings underscore that coordination of the alkene to the third-row transition metal gold(III) can dramatically enhance the acidity of allylic hydrogens, a phenomenon not observed with cobalt, a first-row transition metal, possessing the same oxidation state.

**Table 3:** Calculated Thermodynamic and Kinetic Values for Selected Substituents on 1-Butene Coordinated to Co^III^, Based on the Approach Outlined in Scheme 4b^a^

R^1^	R^2^	Δ*G*_1_	Δ*G*_2_	Δ*G*_3_	Δ*G*^‡^	p*K*_a_
H	H	2.9	29.9	12.1	32.8	8.9
H	CF_3_	5.0	36.0	14.3	41.0	10.5
H	OMe	1.9	32.0	17.4	33.9	12.8
CF_3_	H	3.5	40.1	17.6	43.6	12.9
CF_3_	CF_3_	8.7	42.9	19.2	51.6	14.1
CF_3_	OMe	4.7	39.8	25.0	44.5	18.3
OMe	H	–3.2	46.1	18.6	46.1	16.0
OMe	CF_3_	7.2	46.0	22.0	53.2	16.1
OMe	OMe	3.8	30.6	17.0	34.4	12.5

a Free energies are given in kcal/mol, and the p*K*_a_ values are for the allylic hydrogen.

### Allylic Hydrogen Acidity in 1-Butene Derivatives Coordinated to Rhodium(III) Porphyrin

To further expand our investigation into the relative acidity of allylic hydrogens when coordinated to different transition metals, we next examined rhodium, a second-row transition metal typically exhibiting an oxidation state of +3. The synthesis of rhodium(III) porphyrins has been reported in the literature.^19,20^ Thermodynamic and kinetic results related to the acidity of allylic hydrogens in 1-butene derivatives coordinated to rhodium(III) porphyrin are presented in Table 4. It can be concluded that rhodium(III) exhibits behavior intermediate between gold(III) and cobalt(III). Our results indicate that, within the limited data set used, the formation of the π-complex (**11**) is exergonic for all substrates lacking a CF_3_ substituent. In some cases, such as when both R^1^ and R^2^ are OMe or both are H, the results suggest that under harsh experimental conditions, deprotonation by water may be possible, but the likelihood increases with stronger bases. For none of the substrates studied, is the p*K*_a_ negative, though small positive p*K*_a_ values were observed, indicating that if the intermediate (**12**) is consumed in a subsequent reaction, the pathway involving such a mechanism could be feasible. For instance, we can refer to a study^2^ we reported on alkene isomerization using PdCl_2_ (a second-row transition metal similar to rhodium), where an anionic palladium intermediate with a relative free energy of 11.9 kcal/mol is formed, but this species undergoes further transformations to yield the final product.

**Table 4:** Calculated Thermodynamic and Kinetic Values for Selected Substituents on 1-Butene Coordinated to Rh^III^, Based on the Approach Outlined in Scheme 4c^a^

R^1^	R^2^	Δ*G*_1_	Δ*G*_2_	Δ*G*_3_	Δ*G*^‡^	p*K*_a_
H	H	–3.9	29.4	1.8	29.4	4.2
H	CF_3_	5.0	29.3	4.4	34.3	3.2
H	OMe	–6.1	32.9	7.5	32.9	10.0
CF_3_	H	3.5	31.8	7.7	35.3	5.6
CF_3_	CF_3_	7.1	36.1	7.0	43.2	5.1
CF_3_	OMe	3.3	31.9	14.8	35.2	10.8
OMe	H	–11.2	41.2	8.1	41.2	14.1
OMe	CF_3_	6.6	37.2	13.0	43.8	9.5
OMe	OMe	–4.2	26.7	6.1	26.7	7.6

a Free energies are given in kcal/mol, and the p*K*_a_ values are for the allylic hydrogen.

### Significant Effect of Gold(III) on the Acidity of Allylic Hydrogens

Based on the results obtained, in most derivatives of 1-butene coordinated to AuCl_3_, the allylic hydrogens exhibit significantly enhanced acidity. This pronounced effect is not observed in analogous complexes with Co(III) and Rh(III). This difference in the behavior of these transition metals is a result of varying degrees of relativistic effects, orbital size, and covalent bonding characteristics.^21−23^ Gold(III) benefits from strong relativistic effects and larger, more diffuse 5d orbitals, leading to stronger metal–ligand interactions and greater stabilization of the positive charge on the β-carbon, which facilitates allylic hydrogen deprotonation. On the other hand, these same characteristics of Gold(III) also contribute to the greater thermodynamic stability of the species after deprotonation, resulting in a lower p*K*_a_. Rhodium(III), with less pronounced relativistic effects and intermediate orbital size, exhibits moderate acidity enhancement, while cobalt(III), with smaller 3d orbitals and weaker interactions, shows the least effect.

To investigate the impact of metal identity on ligand–metal interactions, Natural Bond Orbital (NBO) analysis was conducted for Au(III), Rh(III), and Co(III) complexes, where 1-butene is coordinated to the metal center (Figure S4). The results indicate that electron transfer from 1-butene to the Au(III) complex is 0.513e, to the Rh(III) complex is 0.385e, and to the Co(III) complex is 0.275e. This trend highlights the stronger interaction of the alkene with the Au(III) complex and the weaker bonding in the Co(III) complex. For the Au(I) complex, the electron transfer from 1-butene to the metal is only 0.130e, even though this complex, unlike the others, is in a monocationic (+1) state. This highlights the significant role of the metal oxidation state in governing this phenomenon.

### AuCl_3_-Catalyzed Carbonyl–Olefin Metathesis

**Scheme 5:**
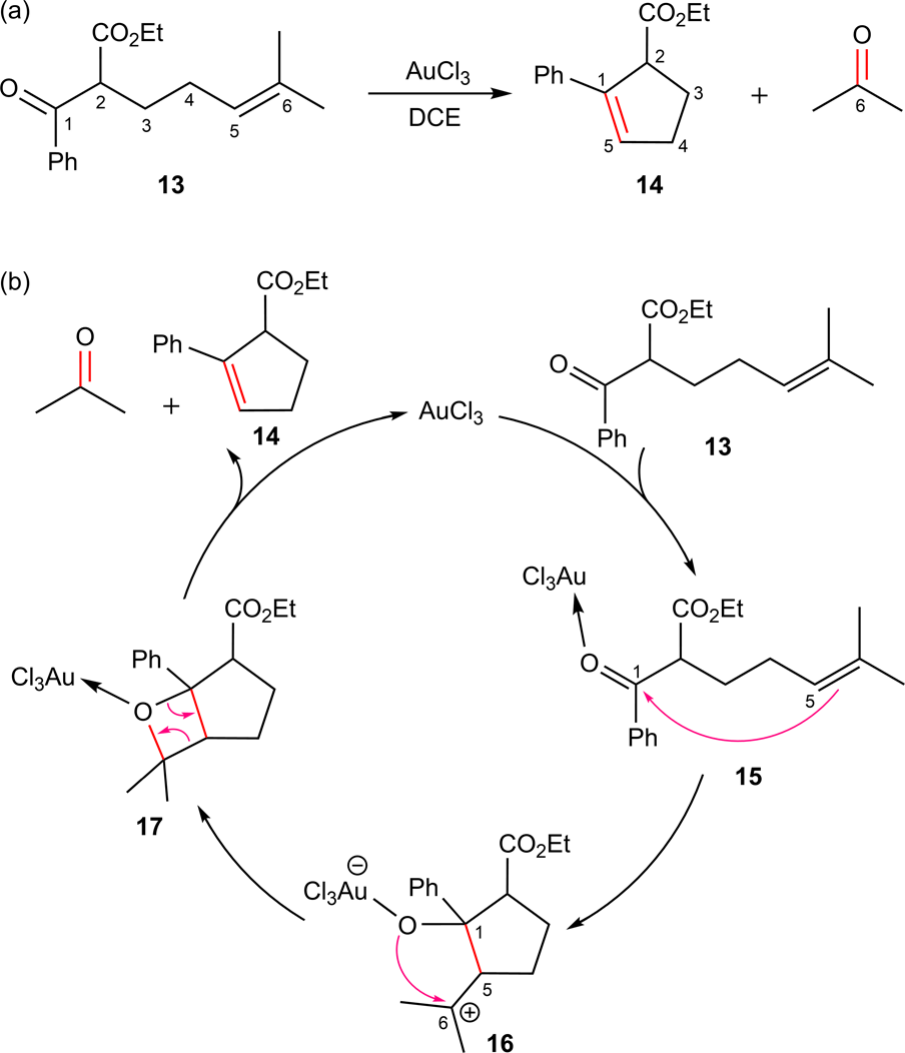
(a) AuCl_3_ Catalyzed Carbonyl–Olefin Metathesis, (b) Proposed Mechanism by Lin and Co-workers

To provide a relevant example of an Au(III) complex rendering allylic hydrogens acidic in an organic transformation, we investigated the AuCl_3_-catalyzed reaction reported by Lin and co-workers,^24^ which features a ring-closing carbonyl–olefin metathesis. Scheme 5a illustrates a representative example of this reaction, for which they conducted detailed DFT calculations. Their proposed mechanism for substrate **13** is outlined in Scheme 5b. According to this mechanism, the reaction is initiated by the coordination of substrate **13** to AuCl_3_ via its carbonyl oxygen (species **15**), leading to an increase in the positive charge on the carbonyl carbon. This induced electron deficiency facilitates the first cyclization, which involves the formation of the C^1^–C^5^ bond and localizes the positive charge on C^6^ (species **16**). A subsequent cyclization, accompanied by C^6^–O bond formation, occurs via nucleophilic attack by the carbonyl oxygen on C^1^ (species **17**). The reaction concludes with the elimination of acetone, yielding the final product (**14**) and regenerating the catalyst.

**Figure 4:**
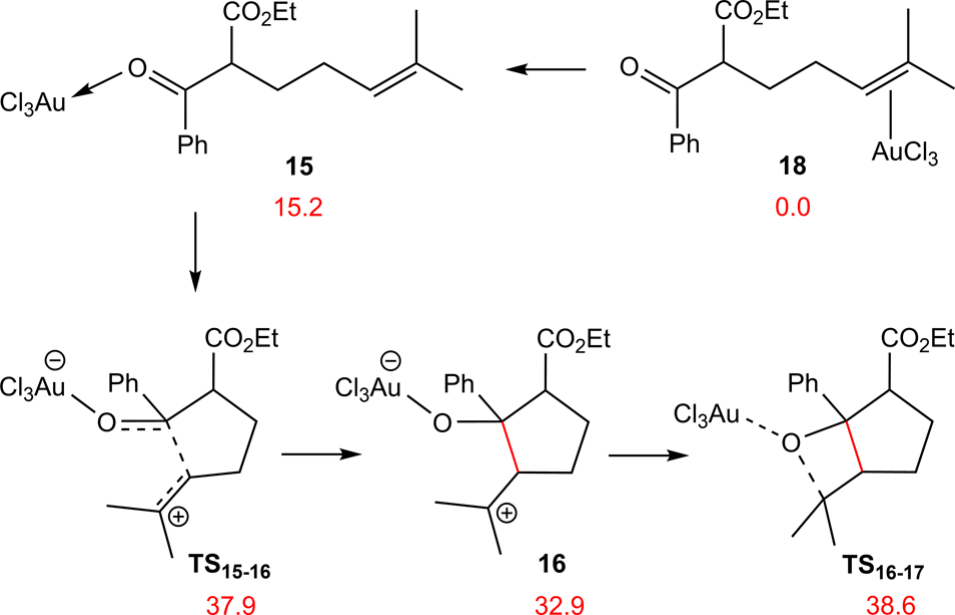
Calculated mechanism for carbonyl–olefin metathesis catalyzed by AuCl_3_, based on the catalytic cycle given in Scheme 5. Free energies are given in kcal/mol.

Our calculations reveal that the coordination of **13** through the π-bond (**18**), where the double bond is significantly polarized due to the presence of two methyl groups on C^6^, is 15.2 kcal/mol more stable than **15**. Consequently, the resting state of the catalyst is the **18**. Figure 4 depicts the relative free energy of key structures from the reaction pathway proposed by Lin et al., as derived from our DFT calculations.

**Figure 5:**
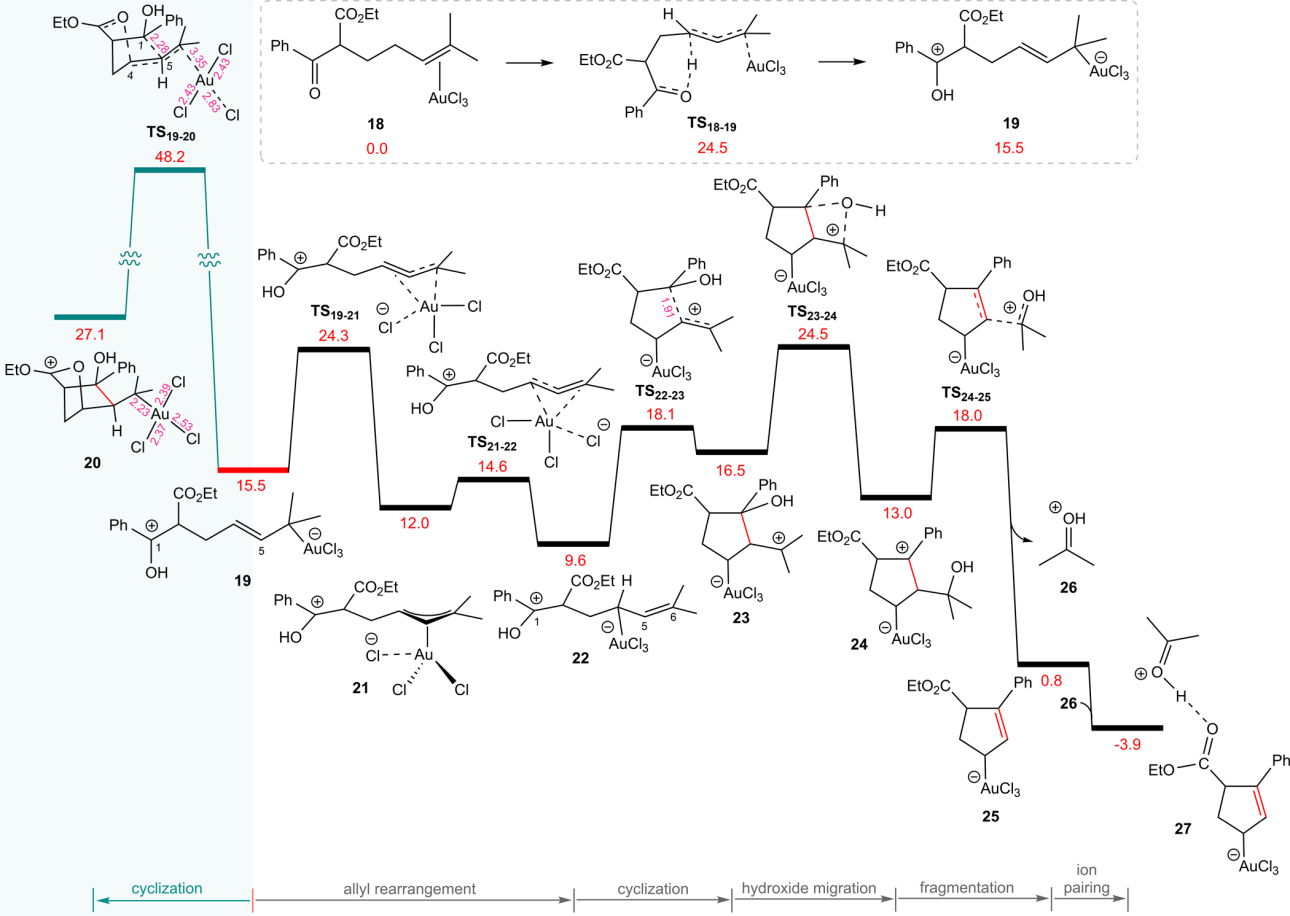
Calculated reaction free energies for carbonyl–olefin metathesis of substrate **13** catalyzed by AuCl_3_, based on the proposed mechanism initiated by allylic hydrogen deprotonation. The relative Gibbs free energy values are given in kcal/mol, and selected distances are provided in (Å).

Our DFT results indicate that the initial cyclization (**TS**_**15–16**_), based on the suggested mechanism, is associated with a prohibitively high free energy barrier of 37.9 kcal/mol, leading to the formation of the highly unstable intermediate **16** with a relative free energy of 32.9 kcal/mol. The subsequent cyclization (**TS**_**16–17**_) is even more challenging, with an activation energy of 38.6 kcal/mol. These results strongly suggest that proceeding through this reaction pathway is not viable. However, our earlier findings suggest that the allylic hydrogen in Au^III^ complex **18** becomes significantly acidic. In this context, the carbonyl oxygen assumes the role of a proton acceptor. As illustrated in Figure 5, the oxygen abstracts the allylic proton, overcoming an activation energy barrier of 24.5 kcal/mol via **TS**_**18–19**_, leading to the formation of intermediate **19** with an overall endergonicity of 15.5 kcal/mol.

The C^1^–C^5^ coupling from species **19** via **TS**_19–20_ exhibits a significantly high energy barrier of 48.2 kcal/mol. Notably, in the corresponding transition structure, concomitant with C^1^–C^5^ bond formation and the localization of positive charge on C^4^, the ester oxygen interacts with C^4^ via its lone pair. Furthermore, a detailed analysis of this transition structure reveals that the gold center approaches a gold(I)-like state; however, this is short-lived, as the gold center in the resulting species (**20**) exhibits a +3 oxidation state.

The η^1^-allyl complex **19** undergoes a downhill rearrangement to another η^1^-allyl complex (**22**) through an η^3^-allyl complex (**21**). The calculated energy barriers for the corresponding transition states **TS**_**19**–**21**_ (η^1^ → η^3^) and **TS**_**21**–**22**_ (η^3^ → η^1^) are 24.3 and 14.6 kcal/mol, respectively. Cyclization from **22** proceeds through the transition structure **TS**_**22**–**23**_, affording intermediate **23**. This cyclization is significantly more facile than that from **19**, due in part to the 5.9 kcal/mol greater stability of **22** relative to **19**, but primarily due to the formation of a more stable carbocation (tertiary vs secondary). Additionally, the results indicate that the interaction between the ester oxygen and the positively charged carbon is insufficient to lower the activation energy of **TS**_**19**–**20**_. It is worth noting that cyclization between C^1^ and C^6^ in **22**, which would result in a secondary carbocation, is also associated with a high energy barrier of 37.4 kcal/mol, rendering it noncompetitive.

Nucleophilic migration of the hydroxide to the positively charged carbon in species **23**, via transition structure **TS**_**23**–**24**_ (Δ*G*^‡^ = 24.5 kcal/mol), affords the more stable species **24**. Subsequently, **24** can undergo fragmentation via transition structure **TS**_**24**–**25**_ (Δ*G*^‡^ = 18.0 kcal/mol), resulting in the formation of η^1^-allyl gold(III) anion **25** and oxonium cation **26**. Cation **26**, owing to its high acidity, forms a strong hydrogen bond with the ester carbonyl oxygen in anion **25**. This interaction results in the formation of a stable adduct (**27**) with a relative free energy of 3.9 kcal/mol.

**Figure 6:**
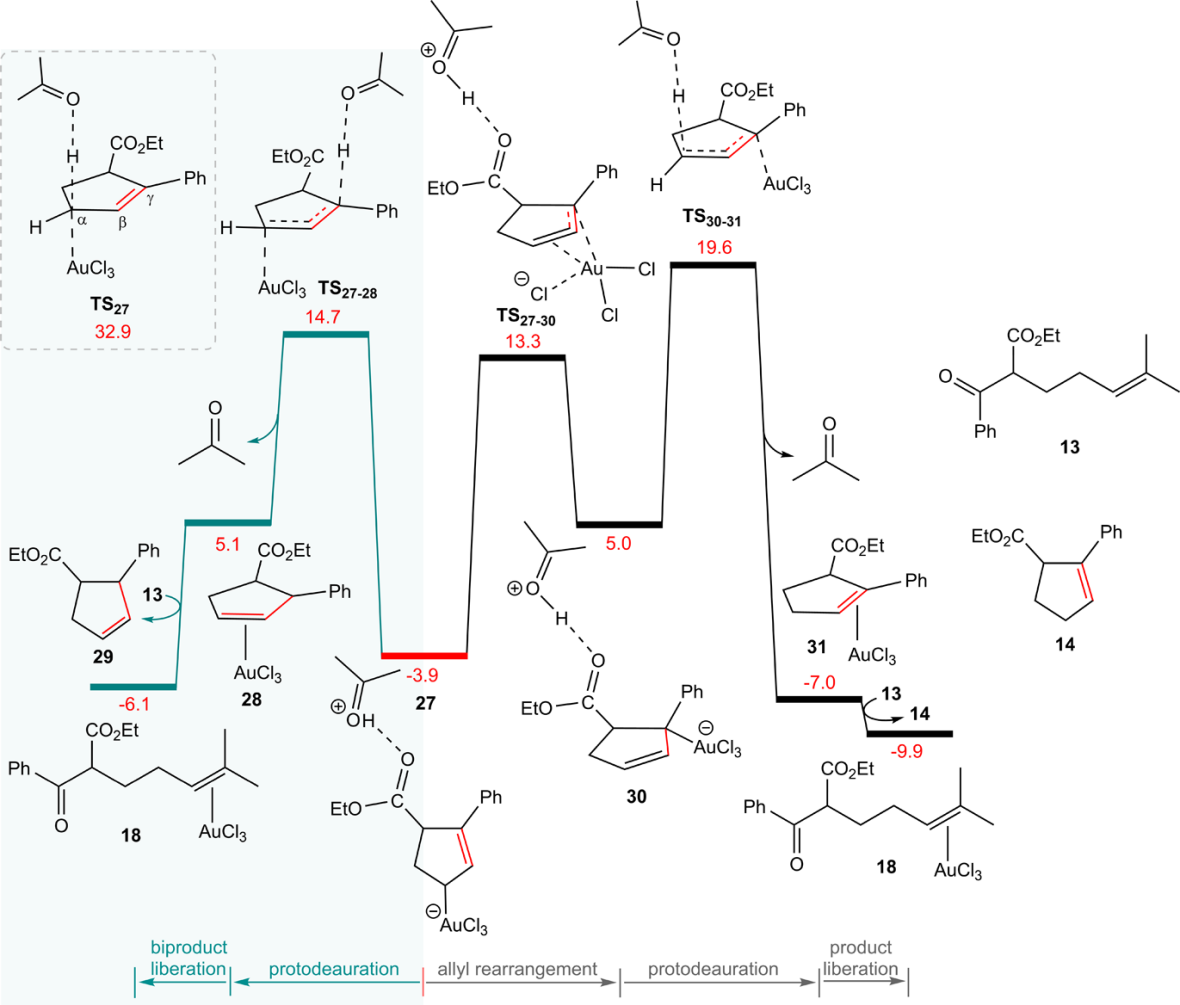
Potential protodeauration (protodemetalation) pathways starting from species **27**. Free energies are given in kcal/mol.

Protodemetalation (referred to as protodeauration in the case of gold) at the α-position in structure **27** yields the final product (Figure 6). However, due to the significant electron deficiency at the C^α^ induced by Au(III), it is insufficiently nucleophilic to effectively attract a proton.^2,25^ This is supported by DFT calculations, which indicate that an overall activation free energy of 36.8 (32.9 – (−3.9)) kcal/mol is required for this process via **TS**_27_. Nevertheless, the γ-position of the η^1^-allyl gold complex is more susceptible to the protodeauration step.

Protodeauration of species **27**, proceeding through transition state **TS**_**27**–**28**_ with an overall activation free energy of 18.6 kcal/mol, produces the side product **29**. Although analogs of this byproduct have been reported for certain substrates,^24^ only product **14** has been observed for substrate **13**. The η^1^-allyl complex **27** can undergo an endergonic rearrangement via transition state **TS**_**27**–**30**_ to form another η^1^-allyl isomer (**30**). Our results suggest that this AuCl_3_ migration occurs without forming a local minimum η^3^-allyl intermediate, directly connecting the η^1^ complexes **27** and **30** through **TS**_**27**–**30**_.

Protodeauration of the rearranged η^1^-allyl complex, occurring at **TS**_**30**–**31**_, leads to complex **31**, in which the experimental product is π-bound to the gold center, with a relative free energy of −7.0 kcal/mol. Substrate substitution liberates the product (**14**) and regenerates complex **18** to initiate the next catalytic cycle. Overall, it can be inferred that **29** is the kinetic product, while **14** is the thermodynamic product of this transformation. Over time, as the energy barrier becomes accessible, thermodynamic control eventually leads to the complete conversion of **29** to the more stable product **14**, with a calculated free energy barrier of 25.7 (19.6 – (−6.1)) kcal/mol.

## Conclusions

The results of this study reveal important insights into the acidity of allylic hydrogens in transition metal-coordinated alkenes, highlighting the significant influence of the nature of the transition metal, its oxidation state, and vinyl substituent effects.^4^ Gold(III), by virtue of its electronic characteristics, dramatically enhances acidity for coordinated alkenes, enabling more efficient proton abstraction. In contrast, rhodium(III) exhibits moderate acidity enhancement, while cobalt(III)-coordinated alkenes display the weakest thermodynamic and kinetic properties associated with allylic hydrogen acidity. AuCl_3_-coordinated alkenes generally exhibit negative p*K*_a_ values and low activation energies for deprotonation, although electron-donating substituents on the vinylic carbons somewhat reduce this acidity. Moreover, the results indicate that, unlike gold(III), gold(I) is unable to induce significant acidity in the allylic hydrogens of coordinated alkenes.

**Scheme 6:**
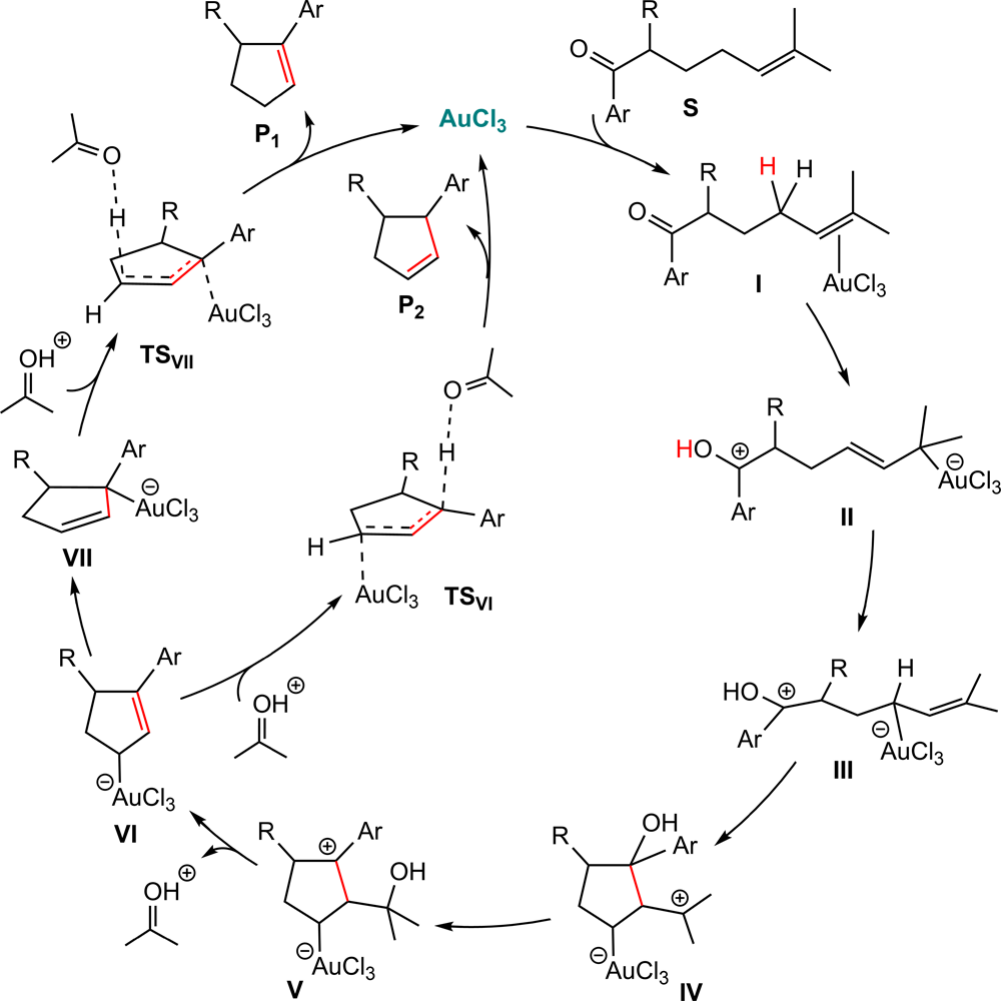
Our Proposed Catalytic Cycle for the Carbonyl–Olefin Metathesis Reaction Catalyzed by AuCl_3_

Besides, we interrogated the mechanism of a carbonyl–olefin metathesis reaction catalyzed by AuCl_3_ using this approach, demonstrating the potential of this previously overlooked perspective for interpreting relevant organometallic transformations. The catalytic cycle for this reaction is depicted in Scheme 6.

These findings broaden our understanding of metal-catalyzed processes and offer valuable insights for designing new catalysts for efficient organic synthesis, particularly for Au(III)-coordinated systems, where significant acidity modulation is observed. Future research could expand upon these results by exploring a wider range of metals and substituents to develop a more generalized understanding of these effects. Additionally, while we employed water as a weak base in our calculations, the presence of stronger bases such as amines or even alcohols could further facilitate the deprotonation pathway for allylic hydrogens in alkenes coordinated to various transition metals, potentially opening new avenues for reactivity and catalyst design.

## Computational Details

Gaussian 16^26^ was used to fully optimize all the structures reported in this paper at the M06 level of theory.^27^ For all the calculations, solvent effects were considered using the SMD solvation model.^28^ The SDD basis set^29,30^ with effective core potential (ECP) was chosen to describe gold, cobalt and rhodium. The 6-31G(d) basis set was used for other atoms.^31^ This basis set combination will be referred to as BS1. Frequency calculations were carried out at the same level of theory as those for the structural optimization. Transition structures were located using the Berny algorithm. IRC calculations were used to confirm the connectivity between transition structures and minima.^32,33^ To further refine the energies obtained from the SMD/M06/SDD,6-31G(d) calculations, we carried out single-point energy calculations using the M06 functional method with the SMD solvation model in water along with a larger basis set (BS2) for all the optimized structures. BS2 utilizes the def2-TZVP basis set^34^ on all atoms. The tight convergence criterion and ultrafine integral grid were exploited to increase the accuracy of the calculations. The free energy for each species in solution was calculated using the following formula:
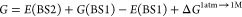
1where Δ*G*^1atm→1M^ = 1.89 kcal/mol is the free-energy change for compression of 1 mol of an ideal gas from 1 atm to the 1 M solution phase standard state. It is worth noting that different spin states have been computed in the energy calculations of nickel complexes, and the most stable ones have been reported.

An additional correction to the Gibbs free energies was made to account for solvent concentration where (H_2_O)_*n*_ is directly involved in transformations. In such cases, the free energy of (H_2_O)_*n*_ is described as follows:
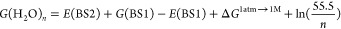
2where the last term corresponds to the free energy required to change the standard state of (H_2_O)_*n*_ from 55.5/nM to 1 M.

The hydration free energy and gas-phase free energy of a proton are well-documented in the literature as −265.9 and −6.3 kcal/mol, respectively.^35−37^ Accordingly, a value of (−265.9) + (−6.3) = −272.2 kcal/mol was used as the free energy of H^+^_solv_ in our calculations when water was used as the solvent.

## Supporting Information Available

Additional computational results and Cartesian coordinates and energy values of the calculated species involved in the carbonyl–olefin metathesis reaction (PDF)

## References

[ref1] Chipman A.; Gouranourimi A.; Farshadfar K.; Olding A.; Yates B. F.; Ariafard A. A computational mechanistic investigation into reduction of gold(III) complexes by amino acid glycine: A new variant for amine oxidation. Chem.—Eur. J. 2018, 24, 8361–8368. 10.1002/chem.201800403. 29655208

[ref2] Farshadfar K.; Chipman A.; Hosseini M.; Yates B. F.; Ariafard A. A modified cationic mechanism for PdCl_2_-catalyzed transformation of a homoallylic alcohol to an allyl ether. Organometallics 2019, 38, 2953–2962. 10.1021/acs.organomet.9b00276.

[ref3] Farshadfar K.; Laasonen K. DFT Mechanistic Investigation into Ni(II)-Catalyzed Hydroxylation of Benzene to Phenol by H_2_O_2_. Inorg. Chem. 2024, 63, 5509–5519. 10.1021/acs.inorgchem.3c04461. 38471975 PMC11186014

[ref4] In this study, we employed the commonly used supporting ligand for each transition metal, which had been synthesized and experimentally characterized. No single ligand could serve as a universal supporting ligand for all the investigated transition metals while simultaneously enabling alkene coordination to the metal center. Clearly, each supporting ligand may influence the extent of allylic hydrogen acidity. However, we were unable to investigate this effect for the transition metal complexes examined in this study.

[ref5] Farshadfar K.; Laasonen K. Comparison of the Efficiency of B–O and B–C Bond Formation Pathways in Borane-Catalyzed Carbene Transfer Reactions Using α-Diazocarbonyl Precursors: A Combined Density Functional Theory and Machine Learning Study. ACS catalysis 2024, 14, 14486–14496. 10.1021/acscatal.4c03368. 39445172 PMC11494835

[ref6] Khakpour R.; Farshadfar K.; Dong S.-T.; Lassalle-Kaiser B.; Laasonen K.; Busch M. Mechanism of CO2 Electroreduction to Multicarbon Products over Iron Phthalocyanine Single-Atom Catalysts. J. Phys. Chem. C 2024, 128, 5867–5877. 10.1021/acs.jpcc.3c08347.

[ref7] Song L.; Tian X.; Farshadfar K.; Shiri F.; Rominger F.; Ariafard A.; Hashmi A. S. K. An unexpected synthesis of azepinone derivatives through a metal-free photochemical cascade reaction. Nat. Commun. 2023, 14, 831 10.1038/s41467-023-36190-z. 36788212 PMC9929248

[ref8] Dasgupta A.; van Ingen Y.; Guerzoni M. G.; Farshadfar K.; Rawson J. M.; Richards E.; Ariafard A.; Melen R. L. Lewis acid assisted Bro̷nsted acid catalysed decarbonylation of Isocyanates: a combined DFT and experimental study. Chem.—Eur. J. 2022, 28, e202201422 10.1002/chem.202201422. 35560742 PMC9541586

[ref9] Farshadfar K.; Bird M. J.; Olivier W. J.; Hyland C. J.; Smith J. A.; Ariafard A. Computational investigation into the mechanistic features of bromide-catalyzed alcohol oxidation by PhIO in water. J. Org. Chem. 2021, 86, 2998–3007. 10.1021/acs.joc.0c02903. 33502190

[ref10] Hu C.; Farshadfar K.; Dietl M. C.; Cervantes-Reyes A.; Wang T.; Adak T.; Rudolph M.; Rominger F.; Li J.; Ariafard A.; et al. Gold-catalyzed [5, 5]-rearrangement. ACS Catal. 2021, 11, 6510–6518. 10.1021/acscatal.1c01108.

[ref11] Krauter C. M.; Hashmi A. S. K.; Pernpointner M. A new insight into gold (I)-catalyzed hydration of alkynes: proton transfer. ChemCatChem. 2010, 2, 1226–1230. 10.1002/cctc.201000136.

[ref12] Hansch C.; Leo A.; Taft R. A survey of Hammett substituent constants and resonance and field parameters. Chem. Rev. 1991, 91, 165–195. 10.1021/cr00002a004.

[ref13] Bordwell F. G. Equilibrium acidities in dimethyl sulfoxide solution. Acc. Chem. Res. 1988, 21, 456–463. 10.1021/ar00156a004.

[ref14] Collado A.; Nelson D. J.; Nolan S. P. Optimizing catalyst and reaction conditions in gold(I) catalysis–ligand development. Chem. Rev. 2021, 121, 8559–8612. 10.1021/acs.chemrev.0c01320. 34259505

[ref15] Zhao Q.; Meng G.; Nolan S. P.; Szostak M. N-Heterocyclic carbene complexes in C–H activation reactions. Chem. Rev. 2020, 120, 1981–2048. 10.1021/acs.chemrev.9b00634. 31967451 PMC7241961

[ref16] Tian X.; Song L.; Farshadfar K.; Rudolph M.; Rominger F.; Oeser T.; Ariafard A.; Hashmi A. S. K. Acyl Migration versus Epoxidation in Gold Catalysis: Facile, Switchable, and Atom-Economic Synthesis of Acylindoles and Quinoline Derivatives. Angew. Chem. 2020, 132, 479–486. 10.1002/ange.201912334. PMC6972584 31622542

[ref17] Sakurai T.; Yamamoto K.; Naito H.; Nakamoto N. The crystal and molecular structure of chloro-α,β,γ,δ-tetraphenylporphinatocobalt(III). Bull. Chem. Soc. Jpn. 1976, 49, 3042–3046. 10.1246/bcsj.49.3042.

[ref18] Adler A. D.; Longo F. R.; Kampas F.; Kim J. On the preparation of metalloporphyrins. Journal of Inorganic and Nucl. Chem. 1970, 32, 2443–2445. 10.1016/0022-1902(70)80535-8.

[ref19] Thompson S. J.; Brennan M. R.; Lee S. Y.; Dong G. Synthesis and applications of rhodium porphyrin complexes. Chem. Soc. Rev. 2018, 47, 929–981. 10.1039/C7CS00582B. 29188830

[ref20] Zhang L.; Chan K. S. Facile Synthesis of Rhodium(III) Porphyrin Silyls by Silicon- Hydrogen Bond Activation with Rhodium(III) Porphyrin Halides and Methyls. Organometallics 2006, 25, 4822–4829. 10.1021/om0604472.

[ref21] Huheey J. E.; Keiter E. A.; Keiter R. L.; Medhi O. K. Inorganic Chemistry: Principles of Structure and Reactivity; Pearson Education India, 2006.

[ref22] Cotton F. A.; Wilkinson G.; Murillo C. A.; Bochmann M. Advanced Inorganic Chemistry; John Wiley & Sons, 1999.

[ref23] Pyykko P. Relativistic effects in structural chemistry. Chem. Rev. 1988, 88, 563–594. 10.1021/cr00085a006.

[ref24] Wang R.; Chen Y.; Shu M.; Zhao W.; Tao M.; Du C.; Fu X.; Li A.; Lin Z. AuCl3-Catalyzed Ring-Closing Carbonyl–Olefin Metathesis. Chem.—Eur. J. 2020, 26, 1941–1946. 10.1002/chem.201905199. 31867760

[ref25] BabaAhmadi R.; Ghanbari P.; Rajabi N. A.; Hashmi A. S. K.; Yates B. F.; Ariafard A. A theoretical study on the protodeauration step of the gold(I)-catalyzed organic reactions. Organometallics 2015, 34, 3186–3195. 10.1021/acs.organomet.5b00219.

[ref26] Frisch M. J. Gaussian 16. Gaussian, Inc.: Wallingford CT, 2016; https://gaussian.com/gaussian16/, Version 16, Revision A.03.

[ref27] Zhao Y.; Truhlar D. G. The M06 suite of density functionals for main group thermochemistry, thermochemical kinetics, noncovalent interactions, excited states, and transition elements: two new functionals and systematic testing of four M06-class functionals and 12 other functionals. Theor. Chem. Acc. 2008, 120, 215–241. 10.1007/s00214-007-0310-x.

[ref28] Marenich A. V.; Cramer C. J.; Truhlar D. G. Universal solvation model based on solute electron density and on a continuum model of the solvent defined by the bulk dielectric constant and atomic surface tensions. J. Phys. Chem. B 2009, 113, 6378–6396. 10.1021/jp810292n. 19366259

[ref29] Dolg M.; Wedig U.; Stoll H.; Preuss H. Energy-adjusted ab initio pseudopotentials for the first row transition elements. J. Chem. Phys. 1987, 86, 866–872. 10.1063/1.452288.

[ref30] Bergner A.; Dolg M.; Küchle W.; Stoll H.; Preuß H. Ab initio energy-adjusted pseudopotentials for elements of groups 13–17. Mol. Phys. 1993, 80, 1431–1441. 10.1080/00268979300103121.

[ref31] Hariharan P. C.; Pople J. A. The influence of polarization functions on molecular orbital hydrogenation energies. Theor. Chim. Acta. 1973, 28, 213–222. 10.1007/BF00533485.

[ref32] Fukui K. The path of chemical reactions-the IRC approach. Acc. Chem. Res. 1981, 14, 363–368. 10.1021/ar00072a001.

[ref33] Fukui K. Formulation of the reaction coordinate. J. Phys. Chem. 1970, 74, 4161–4163. 10.1021/j100717a029.

[ref34] Weigend F.; Furche F.; Ahlrichs R. Gaussian basis sets of quadruple zeta valence quality for atoms H–Kr. J. Chem. Phys. 2003, 119, 12753–12762. 10.1063/1.1627293.

[ref35] Kelly C. P.; Cramer C. J.; Truhlar D. G. Single-ion solvation free energies and the normal hydrogen electrode potential in methanol, acetonitrile, and dimethyl sulfoxide. J. Phys. Chem. B 2007, 111, 408–422. 10.1021/jp065403l. 17214493 PMC2528251

[ref36] Kelly C. P.; Cramer C. J.; Truhlar D. G. Aqueous solvation free energies of ions and ion- water clusters based on an accurate value for the absolute aqueous solvation free energy of the proton. J. Phys. Chem. B 2006, 110, 16066–16081. 10.1021/jp063552y. 16898764

[ref37] Camaioni D. M.; Schwerdtfeger C. A. Comment on ″Accurate experimental values for the free energies of hydration of H^+^, OH^–^, and *H*_3_O^+^″. J. Phys. Chem. A 2005, 109, 10795–10797. 10.1021/jp054088k. 16863129

