# Supplementary material for: Allylic Hydrogen Acidity of 1-Butene Derivatives Coordinated to Transition Metals—A Mechanistic Insight Including Carbonyl–Olefin Metathesis
Source: Inorg Chem. 2025 Feb 25;64(9):4472–82. doi: 10.1021/acs.inorgchem.4c05297 (PMC11898050; doi:10.1021/acs.inorgchem.4c05297)
Supplement: Supplementary file 1 — ic4c05297_si_001.pdf [file ic4c05297_si_001.pdf]

Allylic Hydrogen Acidity of 1-Butene Derivatives Coordinated to Transition Metals; A Mechanistic Insight Including Carbonyl–Olefin Metathesis

Kaveh Farshadfar,* Zonghang Song, and Kari Laasonen*

Department of Chemistry and Material Science, School of Chemical Engineering, Aalto University, 02150 Espoo, Finland

Email: Kaveh.Farshadfar@Aalto.fi, Kari.Laasonen@Aalto.fi

Content:

Figure S1. Comparison of the Au(III)–β-carbon distance in cases where R^2 is hydrogen versus other substituents. (page S2)

Figure S2. Correlation between the relative free energy of the π-complex (ΔG_1) and the Hammett σ^+_p values of the R^2 substituents, excluding hydrogen, with R^1 = H, CF_3, or OMe. (page S3)

Figure S3. Plot of pK_a versus $\Delta G^‡$ for the data presented in Table 1. Each sample is represented by a diamond, where the left triangle indicates the R^1 substituent (excluding NMe_2), and the right triangle represents the R^2 substituent. (page S4)

Figure S4. Total NPA charge on 1-butene coordinated to the four studied complexes: Au(III), Rh(III), Co(III), and Au(I). (page S5)

Table S1. Cartesian coordinates and total energies of the calculated species involved in the carbonyl–olefin metathesis reaction. (page S5)

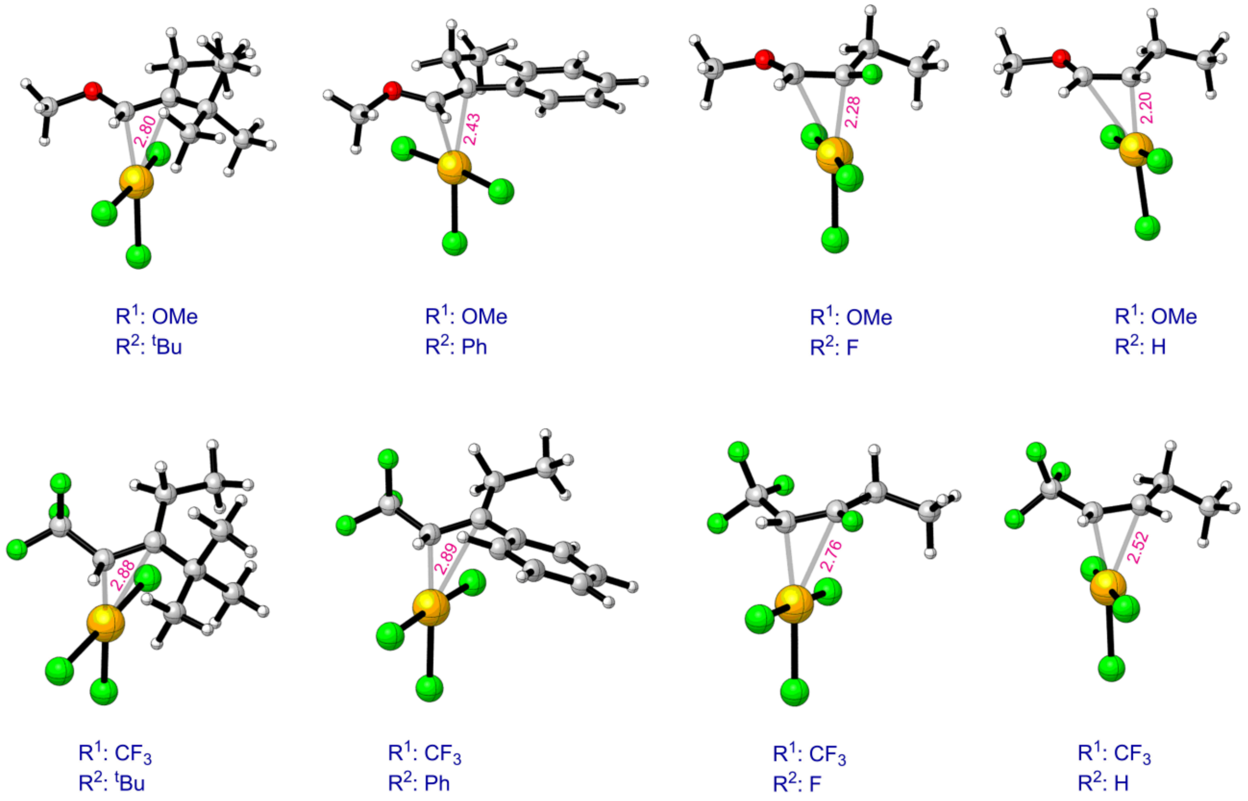

Figure S1. Comparison of the Au(III)–β-carbon distance in cases where R^2 is hydrogen versus other substituents. Distances are given in Å.

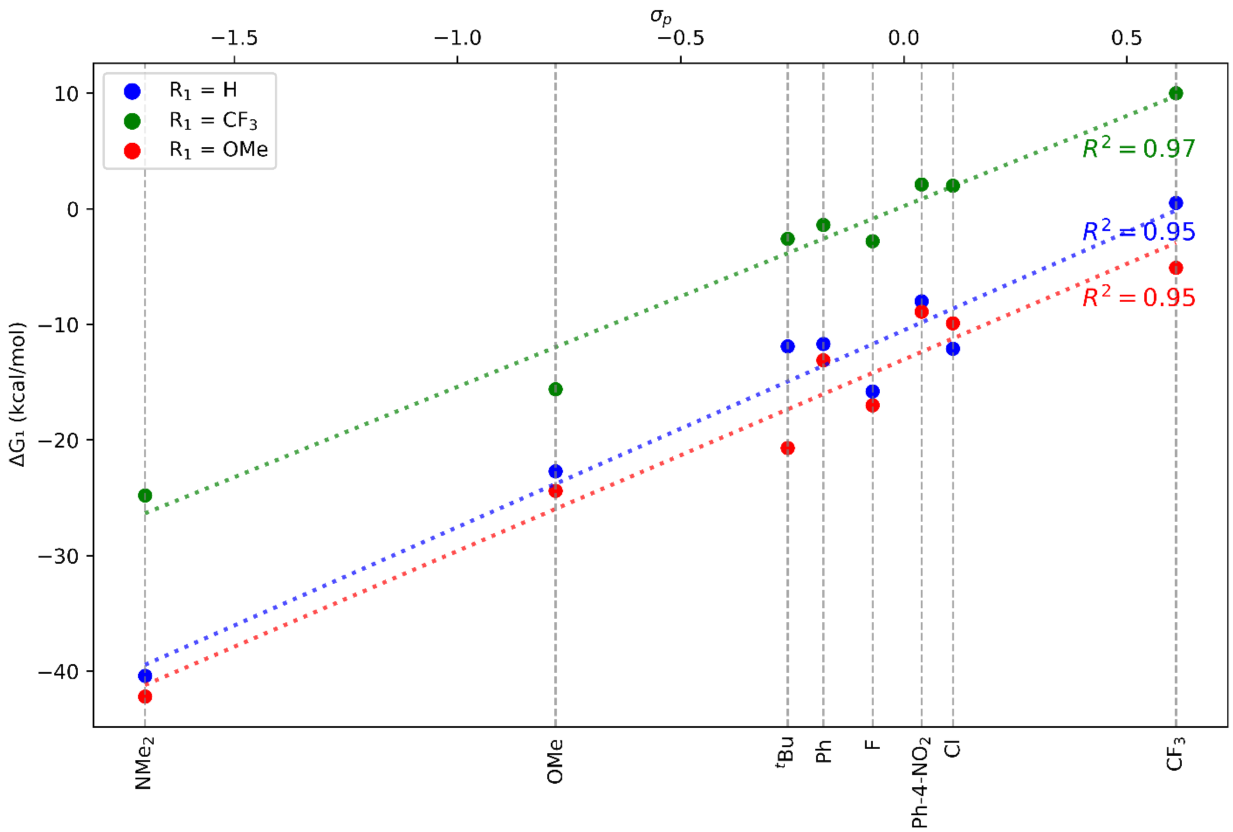

Figure S2. Correlation between the relative free energy of the π-complex (ΔG_1) and the Hammett σ^+_p values of the R^2 substituents, excluding hydrogen, with R^1 = H, CF_3, or OMe

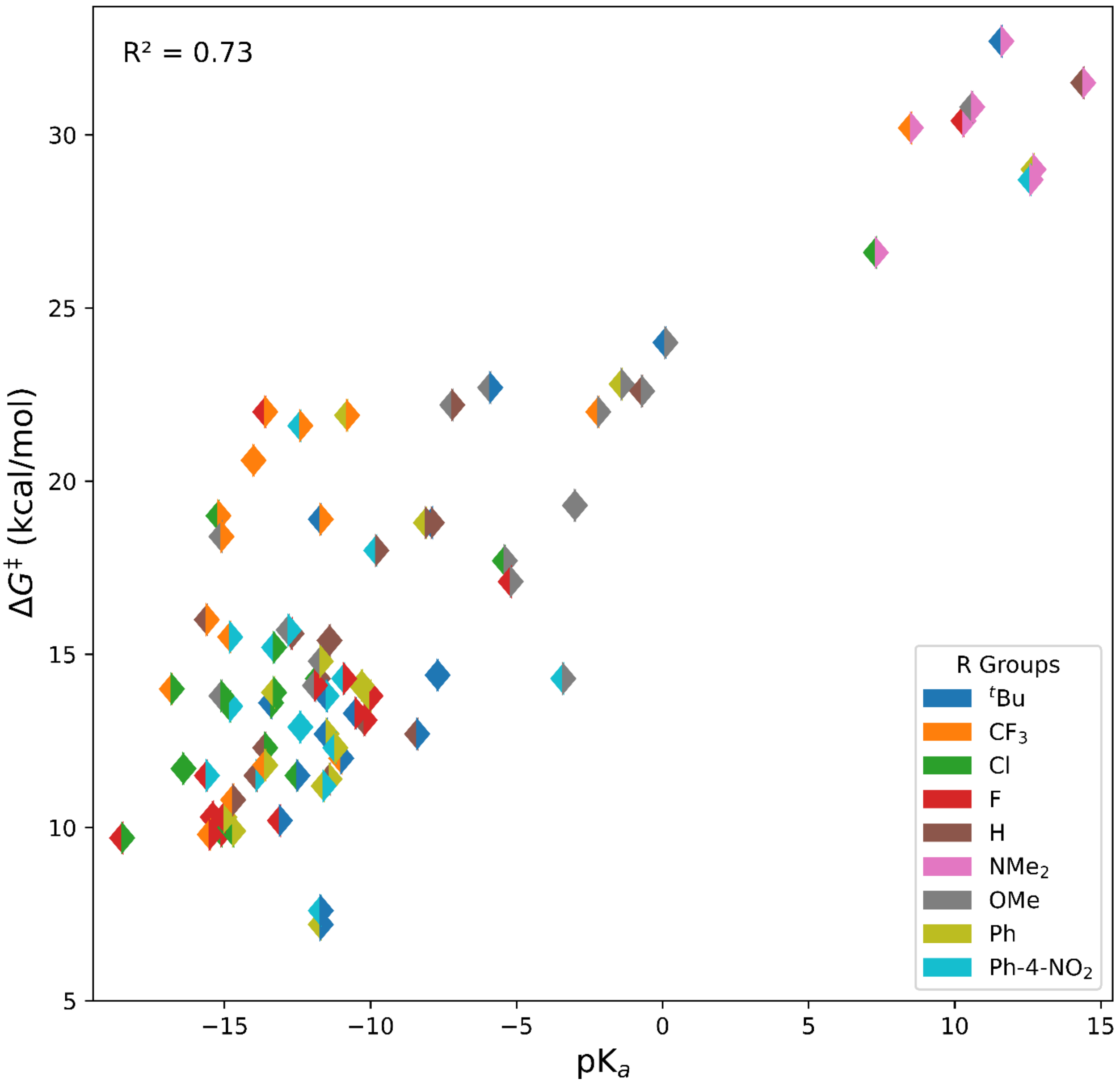

Figure S3. Plot of pK_a versus $\Delta G^{\ddagger}$ for the data presented in Table 1. Each sample is represented by a diamond, where the left triangle indicates the R^1 substituent (excluding NMe_2), and the right triangle represents the R^2 substituent.

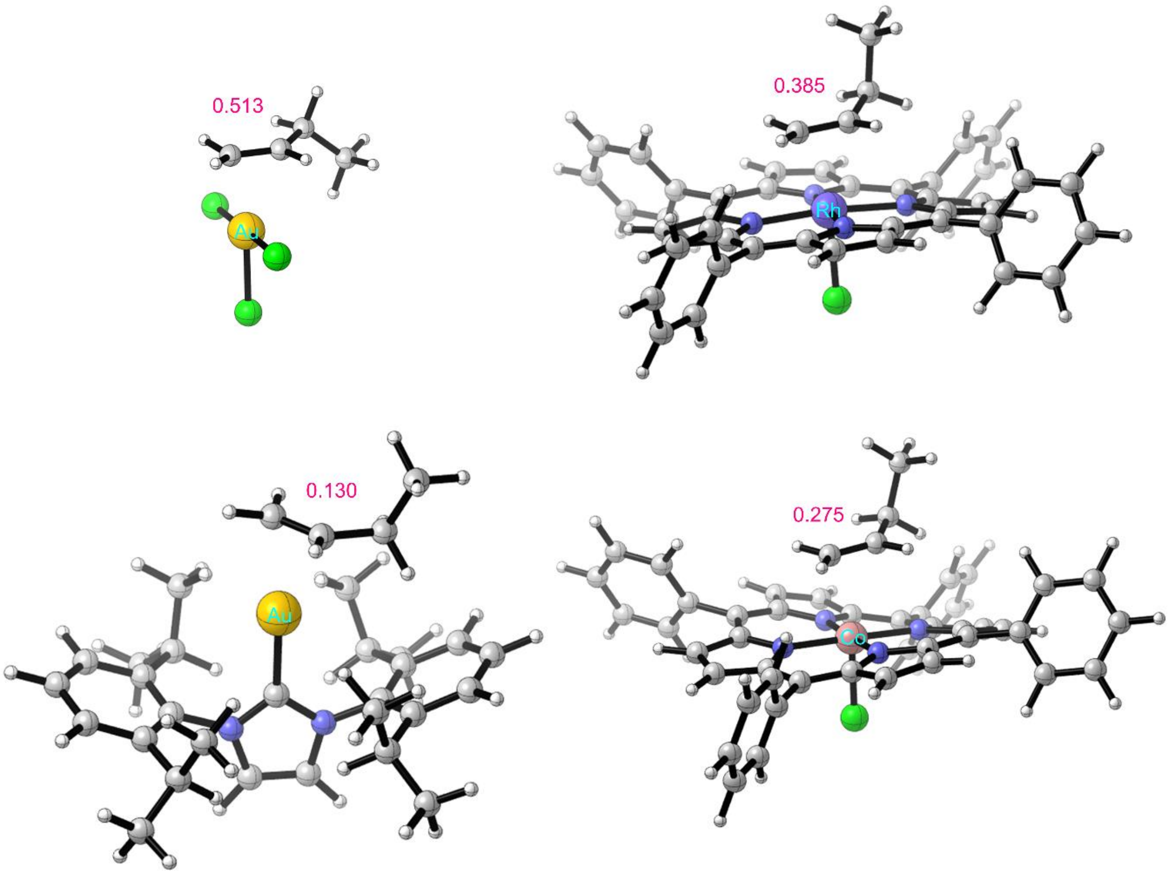

Figure S4. Total natural population analysis (NPA) charge on 1-butene coordinated to the four studied complexes: Au(III), Rh(III), Co(III), and Au(I).

Table S1. Total potential (E), and Gibbs free energies (G) of all structures optimized at the SMD/M06/def2-TZVP level of theory along with the total potential energies calculated by SMD/M06/def2-TZVP//SMD/M06/SDD,6-31G(d) and Cartesian coordinates for the calculated species involved in the carbonyl–olefin metathesis reaction.

13

E (SMD/M06/SDD,6-31G(d)) = -886.118915100

G (SMD/M06/SDD,6-31G(d)) = -885.817629

E (SMD/M06/def2-TZVP//SMD/M06/SDD,6-31G(d)) = -886.463154741

C	-0.29655000	0.34699600	0.16127200
C	-0.09003700	-0.82569000	-0.79986500
C	-1.41156600	-1.54901000	-1.03915700
C	-1.31993200	-2.58586100	-2.15812700
C	-2.61455900	-3.31649300	-2.35841600

C	-3.00235200	-4.42966700	-1.72053400
C	-4.33548700	-5.05875300	-1.98893100
O	-0.91272600	0.14473900	1.19513200
C	-2.17349000	-5.14141500	-0.69447800
H	0.32529200	-0.48009000	-1.75541900
C	0.93702700	-1.73522300	-0.15058900
H	-1.72458400	-2.03072700	-0.10312300
H	-2.18580200	-0.80717300	-1.28697500
H	-1.03548400	-2.07616700	-3.09221900
H	-0.50543300	-3.28834000	-1.93092900
H	-1.95294900	-6.17181700	-1.01473900
H	-1.22424800	-4.64025000	-0.47140900
H	-2.73177800	-5.23021600	0.25042200
H	-4.21995200	-6.09909200	-2.33155200
H	-4.94375200	-5.10508900	-1.07215700
H	-4.90633600	-4.51114200	-2.74972800
O	2.13651200	-1.14697000	-0.14477700
O	0.71394700	-2.82205900	0.33165200
C	3.20473200	-1.87275000	0.49632000
C	4.44154300	-1.02343100	0.40244700
H	2.91587600	-2.07265900	1.53608200
H	3.32451600	-2.83890100	-0.01065100
H	5.28236700	-1.53601200	0.88459300
H	4.70970400	-0.83313400	-0.64415000
H	4.29385700	-0.05890600	0.90378200
C	0.23134500	1.69694500	-0.16663000
C	-0.19825900	2.76809900	0.62856500
C	1.13841900	1.93921900	-1.20451400
C	0.25590100	4.05507100	0.38350200
H	-0.89842500	2.56730100	1.43713900
C	1.60291100	3.22827900	-1.44140200
H	1.51020000	1.12291000	-1.82029800
C	1.15908000	4.28662100	-0.65341100
H	-0.09106000	4.88181300	1.00091400
H	2.31488200	3.40634800	-2.24523400
H	1.51918200	5.29606200	-0.84598100
H	-3.31680100	-2.86733900	-3.06759200

14

E (SMD/M06/SDD,6-31G(d)) = -693.088488238

G (SMD/M06/SDD,6-31G(d)) = -692.862737

E (SMD/M06/def2-TZVP//SMD/M06/SDD,6-31G(d)) = -693.354645214

C	-0.71873400	-0.19495500	-1.83353800
C	0.39799300	-1.20297500	-2.05461400
C	0.14295900	-2.23795200	-0.93980700
C	-1.35141700	-2.12135300	-0.61889700
C	-1.67374300	-0.72766600	-1.05534300

H	0.31107700	-1.66107300	-3.05235300
C	1.77183800	-0.59142900	-1.92203000
H	0.45687000	-3.24839500	-1.22495800
H	0.72352900	-1.93974500	-0.05591100
O	2.55346800	-0.87741900	-2.96997700
O	2.13412600	0.07292000	-0.97546300
C	3.87628500	-0.31058900	-2.94305100
C	4.58537800	-0.76071900	-4.19017600
H	3.78440300	0.78269600	-2.88929800
H	4.38683600	-0.64747900	-2.03160700
H	5.60167300	-0.34952500	-4.21075800
H	4.65725100	-1.85481100	-4.22779400
H	4.05801900	-0.41738100	-5.08887000
C	-0.74137200	1.12345700	-2.47719500
C	0.03892100	1.38712700	-3.61191300
C	-1.54900600	2.15570900	-1.97605600
C	0.00017700	2.63198700	-4.23281200
H	0.67548900	0.60584800	-4.02759500
C	-1.58786300	3.39701500	-2.59613100
H	-2.13899600	1.98353700	-1.07616900
C	-0.81392200	3.64183400	-3.72987200
H	0.61117000	2.81125300	-5.11646900
H	-2.21975900	4.18396500	-2.18647600
H	-0.84122400	4.61732100	-4.21286100
H	-2.61702900	-0.23797700	-0.81557700
H	-1.95162300	-2.85029300	-1.18888500
H	-1.57033300	-2.30611800	0.44202100

15

E (SMD/M06/SDD,6-31G(d)) = -2402.43174871

G (SMD/M06/SDD,6-31G(d)) = -2402.132032

E (SMD/M06/def2-TZVP//SMD/M06/SDD,6-31G(d)) = -2402.87565485

C	-0.14013600	0.12608800	0.11099900
C	0.41290300	-1.02381700	0.91703300
C	1.78929000	-0.80766700	1.57085900
C	2.44561400	-2.12548900	1.99701900
C	2.59878600	-3.11290900	0.87654600
C	3.63467100	-3.20541200	0.02940500
C	3.66113100	-4.23598100	-1.05736700
O	-0.85755600	-0.11222700	-0.89332000
C	4.84422100	-2.32284900	0.07432100
H	0.54925200	-1.83968900	0.18881200
C	-0.61743600	-1.54715600	1.91074500
H	1.71522600	-0.13928100	2.43768900
H	2.43078800	-0.30795500	0.82911800
H	1.85473600	-2.57960700	2.80457000
H	3.42086400	-1.87250300	2.43570300

H	5.76281900	-2.92655800	0.13287700
H	4.84607000	-1.62075100	0.91604100
H	4.92446400	-1.73493500	-0.85304700
H	4.51112600	-4.92372300	-0.92554400
H	3.79757000	-3.76696300	-2.04407800
H	2.73957700	-4.83131600	-1.08635800
O	-1.43420300	-0.58051200	2.31701400
O	-0.65486600	-2.69530600	2.28341600
C	-2.48363800	-0.95442300	3.24151200
C	-3.36297600	0.25062000	3.41792300
H	-2.01313100	-1.27534300	4.17922100
H	-3.02377700	-1.80948600	2.81677300
H	-4.16863200	0.02065000	4.12483300
H	-3.81667300	0.54544700	2.46331900
H	-2.79271700	1.10201000	3.81056700
C	0.12885700	1.53515800	0.38484200
C	0.14816500	2.43450100	-0.69483400
C	0.31904700	2.01068300	1.69253300
C	0.38489900	3.78096200	-0.47011600
H	0.00423700	2.06218500	-1.70673600
C	0.52604500	3.36446800	1.90870000
H	0.25152900	1.33582800	2.54150200
C	0.57026200	4.24653300	0.83065900
H	0.42154600	4.47169800	-1.30968700
H	0.65259600	3.73426900	2.92383600
H	0.74751500	5.30608200	1.00617300
Au	-1.41942700	-2.03900400	-1.55306600
Cl	-3.39678700	-1.82501900	-0.30983300
Cl	-2.09425100	-4.09436600	-2.34730000
Cl	0.57904000	-2.16664900	-2.77924500
H	1.76724400	-3.81138900	0.73435600

TS_{15-16}

E (SMD/M06/SDD,6-31G(d)) = -2402.40156543

G (SMD/M06/SDD,6-31G(d)) = -2402.100821

E (SMD/M06/def2-TZVP//SMD/M06/SDD,6-31G(d)) = -2402.84058711

C	0.32648600	0.68907400	-2.25877600
C	-0.20002800	1.54001500	-1.27253700
C	0.14528500	2.88890100	-1.30158600
C	1.02605300	3.36807200	-2.26842900
C	1.56739200	2.51433000	-3.22236200
C	1.20728500	1.16999100	-3.21986500
C	-1.09627000	0.80616500	-0.29249900
C	-2.51654400	0.51965800	-0.84917900
C	-3.54665500	1.59429200	-0.48156600
C	-2.85736100	2.79543500	0.16985700
C	-1.76390300	2.28472900	1.06619200

C	-2.02973900	1.61875900	2.24877400
C	-0.91434200	1.09622400	3.06257500
O	-0.56415300	-0.17245800	0.35911700
Cl	2.16247600	1.47654700	1.19176000
C	-3.39795800	1.28539100	2.71516900
Cl	0.81024300	-2.77022600	-0.32021300
Cl	3.68033300	-1.35297100	0.51675700
C	-2.91995700	-0.87605900	-0.40629400
O	-2.52820700	-1.77932400	-1.29976000
O	-3.50097300	-1.13675500	0.62491600
C	-2.78083800	-3.16499700	-0.98217100
C	-4.18800600	-3.55486200	-1.35579700
H	0.03534200	-0.36253000	-2.27828600
H	-0.23619400	3.59094500	-0.56547200
H	1.29414600	4.42292600	-2.26347500
H	2.26016000	2.89582500	-3.97005400
H	1.60572300	0.48847600	-3.96924400
H	-2.37922300	0.48682100	-1.93825600
H	-4.11455400	1.89862500	-1.36823400
H	-4.26515600	1.15155500	0.21866100
H	-2.43274500	3.45112100	-0.60026400
H	-3.59221500	3.39833900	0.72256700
H	-0.77586100	2.74851400	1.04051200
H	-1.14413400	1.10482600	4.13465900
H	-0.77820800	0.03552800	2.77258400
H	0.03603500	1.60429300	2.87049800
H	-3.43462500	1.25597800	3.81051200
H	-4.15867800	1.98237900	2.34466000
H	-3.66508800	0.27948000	2.35107900
H	-2.03773500	-3.71574700	-1.56729700
H	-2.57828600	-3.32334200	0.08352900
H	-4.33542600	-4.62714300	-1.17710300
H	-4.92390000	-3.00474100	-0.75743600
H	-4.37871700	-3.35632300	-2.41789200
Au	1.47477600	-0.63695200	0.41356300

16

E (SMD/M06/SDD,6-31G(d)) = -2402.41435640
G (SMD/M06/SDD,6-31G(d)) = -2402.108628
E (SMD/M06/def2-TZVP//SMD/M06/SDD,6-31G(d)) = -2402.85348449

C	-0.68562500	-1.19815000	-1.59360500
C	-1.00721400	0.16205700	-1.57694700
C	-1.31046900	0.77844100	-2.79679700
C	-1.24111100	0.07397900	-3.99469800
C	-0.87538300	-1.26898800	-3.99959300
C	-0.60797900	-1.90404800	-2.79165400
C	-1.12203300	0.92772400	-0.25824200

C	-2.64550600	1.04797800	0.16361900
C	-2.73223800	2.35353600	0.92297000
C	-1.89620900	3.28672000	0.06562500
C	-0.67405500	2.44242600	-0.38614700
C	0.56255800	2.91134600	0.36155800
C	1.10005600	4.21727600	-0.17401300
O	-0.56798000	0.25163800	0.83073600
Cl	2.02246700	1.67708100	-0.22719400
C	0.57393600	2.86067300	1.86466400
Cl	0.62558200	-2.30943500	1.91223200
Cl	3.59568200	-1.13132300	0.88238400
C	-3.10273800	-0.15880100	0.94415800
O	-3.24924100	-1.21619600	0.14072000
O	-3.31570500	-0.16976400	2.13596700
C	-3.54777300	-2.47767000	0.76629800
C	-5.03376500	-2.65568200	0.94500400
H	-0.52144100	-1.72873700	-0.65759800
H	-1.62802500	1.82142700	-2.82608900
H	-1.47726600	0.58122300	-4.92899200
H	-0.81491600	-1.81954900	-4.93691500
H	-0.34754400	-2.96139800	-2.77319300
H	-3.23103200	1.11005400	-0.76598300
H	-3.76641100	2.69810000	1.04625000
H	-2.30139300	2.22771600	1.92605100
H	-2.46267200	3.57220000	-0.83247700
H	-1.62615200	4.21784200	0.57797600
H	0.38072500	5.01023700	0.07171200
H	2.05352700	4.47822700	0.29982900
H	1.22392100	4.19946000	-1.26301700
H	1.56433700	3.12020600	2.25632300
H	-0.12807400	3.62981900	2.21906000
H	0.24958000	1.89747800	2.26627500
H	-3.12838000	-3.22678100	0.08484100
H	-3.01322800	-2.53301700	1.72215500
H	-5.24557700	-3.65066700	1.35596100
H	-5.43810600	-1.90654600	1.63635900
H	-5.55569600	-2.56410400	-0.01582600
Au	1.35432700	-0.39254800	0.81005100
H	-0.46185800	2.65495100	-1.43980300

TS_{16-17}

E (SMD/M06/SDD,6-31G(d)) = -2402.40602880

G (SMD/M06/SDD,6-31G(d)) = -2402.103242

E (SMD/M06/def2-TZVP//SMD/M06/SDD,6-31G(d)) = -2402.84149555

C	0.04291300	0.69018100	-2.19868000
C	-0.31011400	1.62268800	-1.21193900
C	0.08874500	2.94621600	-1.37802300

C	0.85915900	3.32426400	-2.47751700
C	1.24397000	2.38360000	-3.42458800
C	0.82479900	1.06156800	-3.28528800
C	-1.11077400	1.09077800	-0.02712200
C	-2.53528000	0.63811700	-0.57901400
C	-3.58604600	1.58529900	0.00906500
C	-2.81115300	2.77424900	0.57468800
C	-1.47267200	2.21614900	1.04202500
C	-1.44626200	1.55233500	2.36530200
C	-0.21576300	1.57030800	3.13779500
O	-0.48614100	0.03152900	0.63990200
Cl	2.43795100	1.47961800	0.75922400
C	-2.61393900	0.93358800	2.96510400
Cl	0.49178700	-2.73126600	-0.10544900
Cl	3.57280400	-1.51018300	-0.02714800
C	-2.81325800	-0.81452800	-0.26417600
O	-2.54581400	-1.59090000	-1.31010100
O	-3.25504300	-1.22739400	0.79115100
C	-2.79150300	-3.00549100	-1.16130100
C	-4.23184500	-3.32731400	-1.46806600
H	-0.30546400	-0.34103600	-2.11637400
H	-0.18263900	3.71100400	-0.65206600
H	1.16397100	4.36421700	-2.58335000
H	1.85403900	2.67908000	-4.27645000
H	1.09664600	0.31656000	-4.03145300
H	-2.51450600	0.73364000	-1.66878400
H	-4.31186000	1.90063800	-0.74882200
H	-4.15342900	1.07161100	0.79407000
H	-2.60300800	3.50458700	-0.21898200
H	-3.35436600	3.30678800	1.36556500
H	-0.09676000	0.65851900	3.73718700
H	0.67730600	1.81059200	2.55467400
H	-0.37938200	2.38688400	3.87144500
H	-3.51623200	1.54805800	2.84671900
H	-2.82201700	0.01453300	2.37081500
H	-2.45511000	0.65328800	4.01016900
H	-2.10816500	-3.47744000	-1.87432600
H	-2.50893900	-3.30868300	-0.14754500
H	-4.38815700	-4.41218800	-1.42123000
H	-4.90586700	-2.85448800	-0.74339800
H	-4.50356700	-2.98611800	-2.47482300
Au	1.42857300	-0.60801700	0.28854700
H	-0.66898700	2.96366300	1.06028100

18

E (SMD/M06/SDD,6-31G(d)) = -2402.45546091

G (SMD/M06/SDD,6-31G(d)) = -2402.157580

E (SMD/M06/def2-TZVP//SMD/M06/SDD,6-31G(d)) = -2402.89806264

C	-0.09283400	-0.08290800	-0.25540600
C	0.27288700	-1.29348100	0.61199600
C	-0.85032600	-2.32396000	0.65401200
C	-2.05004400	-1.91069600	1.51387400
C	-2.67275000	-0.61912700	1.05489800
C	-3.44488400	-0.49057300	-0.12841500
C	-3.68590700	0.83707400	-0.73504300
O	-0.63765400	-0.28986900	-1.33135200
C	-3.95993600	-1.65074500	-0.87554200
H	0.52010500	-0.98070800	1.63605000
C	1.53411000	-1.90031600	0.01833400
H	-0.44614800	-3.25689500	1.06485200
H	-1.16790800	-2.54426800	-0.37418200
H	-1.71949200	-1.77543700	2.55461000
H	-2.77997300	-2.72894000	1.51944300
H	-4.95929800	-1.45159500	-1.28347600
H	-3.95231900	-2.59057800	-0.32008600
H	-3.28821800	-1.75863000	-1.74599600
H	-4.76596600	1.04294800	-0.78018500
H	-3.34554800	0.79696400	-1.78124200
H	-3.17698200	1.65239100	-0.21205100
O	2.49416500	-0.97830800	-0.06935100
O	1.65806500	-3.05061200	-0.33039700
C	3.75599500	-1.41271100	-0.61908300
C	4.67348700	-0.22227200	-0.62427100
H	3.57900700	-1.80724600	-1.62768800
H	4.14130200	-2.23248900	0.00060400
H	5.64919500	-0.50728100	-1.03512500
H	4.82696800	0.16001400	0.39245500
H	4.26338500	0.58692800	-1.24091800
C	0.18095700	1.29950700	0.21121100
C	-0.28094400	2.35417300	-0.59043200
C	0.83270700	1.59514400	1.41544900
C	-0.10776100	3.67110200	-0.19297700
H	-0.77967800	2.11682000	-1.52829100
C	1.01043300	2.91627500	1.80955100
H	1.22112700	0.80324600	2.05218500
C	0.53642600	3.95362200	1.01113200
H	-0.47396000	4.48176900	-0.82023900
H	1.52043300	3.13597400	2.74548600
H	0.67163300	4.98692400	1.32632700
Au	-4.40846600	-0.07505300	2.34501300
Cl	-5.47195800	-2.15298500	2.04695800
Cl	-6.06450200	0.51057500	3.96099600
Cl	-3.36182800	2.01624900	2.67494600
H	-2.07934800	0.27253900	1.28156900

TS$_{18-19}$

E (SMD/M06/SDD,6-31G(d)) = -2402.41594709

G (SMD/M06/SDD,6-31G(d)) = -2402.122480

E (SMD/M06/def2-TZVP//SMD/M06/SDD,6-31G(d)) = -2402.85454862

C	1.02530900	0.96166700	-1.02920100
C	1.41723900	0.07303400	0.13598300
C	0.21168600	-0.28478800	1.02446400
C	-0.94576500	-0.85676500	0.22555100
C	-2.25834200	-0.55656600	0.57531000
C	-3.46229200	-1.18334800	0.06922000
C	-4.66968300	-1.13374700	0.98063200
O	-0.14249000	0.90431400	-1.46591900
C	-3.31197200	-2.53963300	-0.56833500
H	2.15821800	0.58007100	0.76391700
C	2.04153700	-1.19354600	-0.42903700
H	0.55089200	-0.99754100	1.78827100
H	-0.11615100	0.62329000	1.54948600
H	-0.76139100	-1.80280100	-0.29566900
H	-4.24571300	-2.85021900	-1.05092000
H	-2.50836300	-2.60757900	-1.30492000
H	-3.10230600	-3.25792800	0.24144000
H	-5.59799900	-1.24865400	0.40617500
H	-4.61234800	-1.98894600	1.67260400
H	-4.73102900	-0.21472200	1.56943500
O	2.81884300	-1.77810100	0.47240000
O	1.81913600	-1.61657500	-1.54201400
C	3.42280800	-3.03344800	0.07719900
C	4.24284700	-3.51878200	1.23847800
H	2.62097200	-3.73284900	-0.18998900
H	4.02980700	-2.85517800	-0.81894500
H	4.71919800	-4.47175300	0.97991400
H	5.03075800	-2.79939300	1.49266000
H	3.61610000	-3.67768600	2.12449300
C	1.98502400	1.86042400	-1.65662000
C	1.53360600	2.73296600	-2.66183500
C	3.34320000	1.85461000	-1.30047400
C	2.42255300	3.59072300	-3.28658400
H	0.48121700	2.73028500	-2.93727400
C	4.23007300	2.70767900	-1.93975500
H	3.72138800	1.17889100	-0.53568700
C	3.77024200	3.57754100	-2.92638800
H	2.06904800	4.27089800	-4.05832300
H	5.28299500	2.69675800	-1.66723300
H	4.46844000	4.25096000	-3.42041000
Au	-3.81451200	0.47846900	-1.42677500
Cl	-3.33940100	-0.95820600	-3.24013200

Cl	-4.32066200	2.24466800	-3.06147800
Cl	-4.23575500	2.05850600	0.30996400
H	-2.41156400	0.28793200	1.25488700
H	-0.83616500	0.00398100	-0.73089700

19

E (SMD/M06/SDD,6-31G(d)) = -2402.43848976

G (SMD/M06/SDD,6-31G(d)) = -2402.136279

E (SMD/M06/def2-TZVP//SMD/M06/SDD,6-31G(d)) = -2402.87769565

C	1.09999800	0.79232300	0.32611500
C	1.08321300	-0.68517700	0.10117700
C	-0.24949300	-1.08585300	-0.63071500
C	-0.47818500	-0.39300400	-1.93331100
C	-0.75020700	-0.95396700	-3.12276600
C	-0.71425700	-2.38440900	-3.53187100
C	-1.14064100	-2.60924600	-4.96777700
O	1.71634900	1.54397000	-0.51435700
C	0.63432200	-3.03314100	-3.29778400
H	1.09028300	-1.21195700	1.06052400
C	2.25216200	-1.17027000	-0.72468800
H	-1.07280400	-0.84095400	0.05730100
H	-0.24183100	-2.17867400	-0.72503700
H	-0.55677900	0.69651400	-1.88073700
H	0.65303300	-4.07099800	-3.64866600
H	0.96076100	-3.02101900	-2.25477000
H	1.36737900	-2.45990200	-3.89141300
H	-1.24818800	-3.68020000	-5.18513300
H	-0.34944900	-2.21875500	-5.63103200
H	-2.07740600	-2.10134300	-5.21786600
O	2.52769700	-2.42541700	-0.48841900
O	2.83094000	-0.47258100	-1.55149000
C	3.59466400	-3.04250500	-1.26918600
C	3.53020000	-4.51878900	-1.00869700
H	4.53907600	-2.59065600	-0.94343000
H	3.43573600	-2.79282900	-2.32469500
H	4.32784900	-5.02078300	-1.56845300
H	2.56769100	-4.93379200	-1.33443500
H	3.66550400	-4.74026300	0.05657500
C	0.39605700	1.43193400	1.39423000
C	0.34726900	2.84347600	1.42942400
C	-0.24348000	0.68575000	2.40698600
C	-0.32718400	3.48496400	2.44907400
H	0.83633800	3.41922600	0.64775200
C	-0.91475900	1.34044900	3.42327300
H	-0.21502500	-0.40126500	2.40925500
C	-0.95707500	2.73458500	3.44450800
H	-0.36830800	4.57106500	2.47507800

H	-1.40586400	0.76621400	4.20471800
H	-1.48671100	3.24350300	4.24759700
Au	-2.32105800	-3.31263700	-2.33872600
Cl	-0.89742100	-5.01864900	-1.48238200
Cl	-4.21321000	-4.25675700	-0.94259300
Cl	-3.85027300	-1.69570800	-3.22782400
H	-1.04653300	-0.26899000	-3.92246500
H	2.22649400	0.96390600	-1.17554000

TS_{19-20}

E (SMD/M06/SDD,6-31G(d)) = -2402.39272353
G (SMD/M06/SDD,6-31G(d)) = -2402.093293
E (SMD/M06/def2-TZVP//SMD/M06/SDD,6-31G(d)) = -2402.82285993

C	1.26077000	0.35182200	0.14718400
C	1.36676000	-1.03168200	0.77784200
C	0.17435100	-1.91873500	0.40118700
C	0.47760700	-2.00154900	-1.08042600
C	0.26933100	-0.63605900	-1.65177400
C	0.91888600	-0.04849200	-2.71780500
C	0.40130300	1.22602200	-3.26903600
O	2.45109700	0.78499000	-0.24732200
C	2.22178100	-0.49335100	-3.26992600
H	1.60859800	-0.93889800	1.84476200
C	2.42325600	-1.76910600	0.01136500
H	-0.80303300	-1.47078400	0.59627300
H	0.23504100	-2.89302100	0.89706300
H	0.02427200	-2.81602700	-1.65463900
H	2.35182200	-0.11401500	-4.29055000
H	2.34508100	-1.58016000	-3.27462900
H	3.02859900	-0.04585300	-2.66044800
H	0.17960500	1.08370900	-4.33768600
H	1.19041000	1.99486300	-3.22931800
H	-0.50656500	1.58046700	-2.77212300
O	3.63416500	-1.84879700	0.34868900
O	1.95424300	-2.32111300	-1.04425600
C	4.58808000	-2.60115400	-0.51906100
C	5.91390300	-2.55648700	0.17053700
H	4.57762800	-2.09451400	-1.49010600
H	4.18092600	-3.61203100	-0.62370100
H	6.64226200	-3.09695800	-0.44474000
H	5.86685400	-3.04181600	1.15180800
H	6.26442400	-1.52556800	0.29273300
C	0.24508500	1.30885100	0.56804400
C	0.14371400	2.55313800	-0.07908200
C	-0.60457100	1.02278600	1.64818500
C	-0.78993700	3.48405500	0.34477900
H	0.76693600	2.79017700	-0.94246600

C	-1.52776300	1.96677200	2.07324200
H	-0.52680700	0.08064000	2.18555000
C	-1.62731000	3.19273500	1.42096300
H	-0.87008800	4.43871700	-0.16977700
H	-2.17188000	1.74291300	2.92045900
H	-2.36027700	3.92577000	1.75132900
Au	-1.28602300	-2.06578300	-4.23458200
Cl	0.69016300	-3.47079400	-4.42832500
Cl	-2.82476200	-0.34503800	-3.47012100
Cl	-3.04817600	-3.53995400	-5.88973300
H	2.46200300	1.75092800	-0.41426100
H	-0.73802300	-0.26645500	-1.44765300

20

E (SMD/M06/SDD,6-31G(d)) = -2402.43056977

G (SMD/M06/SDD,6-31G(d)) = -2402.122980

E (SMD/M06/def2-TZVP//SMD/M06/SDD,6-31G(d)) = -2402.86461327

C	1.02386700	0.07302700	-0.25119500
C	1.27905400	-1.10704000	0.78572000
C	0.22592000	-2.18004800	0.45713100
C	0.56512500	-2.17255600	-1.01819600
C	0.32088900	-0.72187300	-1.42148100
C	0.58939200	-0.32296700	-2.87072100
C	0.29206700	1.12903300	-3.17327900
O	2.28161400	0.53834200	-0.70151400
C	1.94617300	-0.69323400	-3.42024800
H	1.45032300	-0.79097300	1.81686000
C	2.43129500	-1.77071200	0.12540900
H	-0.80838600	-1.88805700	0.64942100
H	0.42949600	-3.13985000	0.94446900
H	0.17972700	-2.95321100	-1.67634200
H	2.02000100	-0.42881000	-4.48144400
H	2.20144300	-1.75088300	-3.31580400
H	2.69537800	-0.09986400	-2.87432000
H	0.22533400	1.29365300	-4.25766800
H	1.13469700	1.73966000	-2.80929800
H	-0.62872700	1.49240200	-2.70768600
O	3.63627700	-1.73682400	0.50842800
O	2.05995400	-2.39142500	-0.93874600
C	4.67768800	-2.34797500	-0.35369400
C	5.98421400	-2.15970200	0.35091100
H	4.62104100	-1.82683100	-1.31634300
H	4.40375800	-3.39966200	-0.48708300
H	6.77913600	-2.59820800	-0.26306300
H	5.98539300	-2.66312700	1.32440200
H	6.20831400	-1.09678800	0.49515200
C	0.19004100	1.20384400	0.32194100

C	0.51286200	2.52913900	0.01685400
C	-0.91304200	0.95994000	1.14623200
C	-0.24708200	3.58120600	0.52038800
H	1.36088500	2.74840500	-0.62875600
C	-1.67121600	2.01100100	1.64954100
H	-1.19613400	-0.05610400	1.41347700
C	-1.34203500	3.32725400	1.33824200
H	0.02250200	4.60503300	0.26651600
H	-2.52448600	1.79601900	2.29052000
H	-1.93722300	4.14954700	1.73138700
Au	-1.01520300	-1.42620400	-3.96767700
Cl	0.51398800	-2.78916200	-5.16286400
Cl	-2.67662100	-0.20343600	-2.75256000
Cl	-2.88506200	-2.58287100	-5.21510200
H	2.68145200	1.07544800	0.00712200
H	-0.75186000	-0.57360200	-1.24460100

$TS_{19\text{-}21}$

E (SMD/M06/SDD,6-31G(d)) = -2402.42711164

G (SMD/M06/SDD,6-31G(d)) = -2402.128366

E (SMD/M06/def2-TZVP//SMD/M06/SDD,6-31G(d)) = -2402.86019087

C	0.76059200	0.77326100	0.18246500
C	0.82108100	-0.71745400	0.03793800
C	-0.57635500	-1.27819900	-0.40842200
C	-1.07563700	-0.66740500	-1.67615900
C	-1.22793400	-1.16496800	-2.94848400
C	-1.11480500	-2.52029400	-3.45009100
C	-1.45264200	-2.73127800	-4.89247800
O	1.20465200	1.51287100	-0.76981600
C	-0.09871700	-3.49377500	-2.93780800
H	1.03981900	-1.17190100	1.01034800
C	1.86410600	-1.17874700	-0.95635100
H	-1.28942700	-1.03917800	0.39216300
H	-0.48984300	-2.36785900	-0.43578400
H	-1.37381400	0.38068400	-1.57502300
H	-0.35503000	-4.51396400	-3.24153300
H	0.05796700	-3.48526100	-1.85673200
H	0.85700400	-3.23592100	-3.42566200
H	-1.80595500	-3.75682900	-5.05895100
H	-0.53191800	-2.60699100	-5.48669900
H	-2.21094700	-2.02911900	-5.25231500
O	2.27106600	-2.39318400	-0.70742900
O	2.23308600	-0.49664500	-1.90705700
C	3.27312900	-2.96702900	-1.60068800
C	3.40771300	-4.41665700	-1.23860400
H	4.20087200	-2.40247700	-1.44997100
H	2.94035900	-2.81565800	-2.63397600

H	4.17180000	-4.87821600	-1.87462800
H	2.46360200	-4.95362800	-1.39293200
H	3.71341200	-4.53647800	-0.19250800
C	0.16232400	1.42231200	1.30447600
C	0.02033100	2.82853700	1.28344200
C	-0.29693000	0.68922400	2.42058900
C	-0.57018000	3.47653200	2.34944100
H	0.36870400	3.39290200	0.42212900
C	-0.88674400	1.35061900	3.48118200
H	-0.19083900	-0.39206800	2.46537800
C	-1.02386000	2.73881000	3.44543400
H	-0.68539400	4.55737200	2.33309200
H	-1.24142400	0.78795800	4.34087800
H	-1.49082900	3.25339700	4.28299100
H	-1.65727100	-0.45836300	-3.66030700
H	1.64295000	0.93197300	-1.47883800
Au	-2.99143300	-2.59288400	-2.18519400
Cl	-2.96196600	-5.05492300	-2.15279200
Cl	-4.46774200	-1.19806500	-3.92583600
Cl	-4.42923200	-2.18622000	-0.17021000

21

E (SMD/M06/SDD,6-31G(d)) = -2402.44437529

G (SMD/M06/SDD,6-31G(d)) = -2402.147438

E (SMD/M06/def2-TZVP//SMD/M06/SDD,6-31G(d)) = -2402.87807393

C	-0.33406300	1.93002800	-0.86941600
C	0.48431700	0.74195700	-0.45964100
C	-2.31345800	-1.81741900	-4.13221900
C	-1.40373700	-2.59638200	-3.25233200
C	-0.75350400	-2.08807200	-2.11541700
C	-1.18988300	-0.94785800	-1.38749300
C	-0.45665600	-0.47575100	-0.16964900
O	-0.37105700	2.27720900	-2.10555200
H	1.00712700	0.96318800	0.47881100
C	1.49987300	0.30968200	-1.49389100
H	-3.24014000	-2.37758400	-4.31581200
H	-2.56679800	-0.82083300	-3.76010600
H	-1.18002300	-0.17617200	0.59859600
H	0.14870500	-1.28617200	0.25416300
O	2.39932000	-0.48647900	-0.98706000
O	1.43234400	0.61790300	-2.68097400
C	3.37108500	-1.07683800	-1.90183300
C	4.17155900	-2.06659000	-1.10774700
H	3.98021000	-0.26130100	-2.30859300
H	2.81728900	-1.54346700	-2.72498300
H	4.91601100	-2.53728200	-1.76012400
H	3.52650300	-2.85309000	-0.69746100

H	4.69916900	-1.57680900	-0.28087400
C	-1.15394600	2.65669000	0.04793900
C	-2.06898100	3.60710800	-0.45836100
C	-1.08073000	2.42861400	1.43895600
C	-2.89463700	4.29654000	0.40725800
H	-2.12994200	3.78082300	-1.52978200
C	-1.90970600	3.12872400	2.29498500
H	-0.37153500	1.71635300	1.85425200
C	-2.81648400	4.05627100	1.78081200
H	-3.60671300	5.02034900	0.01880900
H	-1.85370600	2.95375400	3.36628500
H	-3.47170100	4.59903200	2.45945700
H	0.01554000	-2.71373300	-1.65525900
H	0.27658600	1.70049500	-2.63914500
Au	-2.70648000	-2.44685300	-0.95661600
Cl	-4.05872000	-0.82946100	0.35158300
Cl	-0.97588200	-3.74048000	0.90576900
Cl	-4.32370300	-4.25204100	-1.38144700
H	-1.82047600	-1.71111400	-5.11163500
C	-1.05227900	-3.95871200	-3.72149100
H	-0.43012200	-3.87545500	-4.62702900
H	-1.95552300	-4.50848800	-4.01796600
H	-0.50599500	-4.53445900	-2.96635000
H	-1.81475300	-0.20581500	-1.89634000

$TS_{21\text{-}22}$

E (SMD/M06/SDD,6-31G(d)) = -2402.44434382

G (SMD/M06/SDD,6-31G(d)) = -2402.143784

E (SMD/M06/def2-TZVP//SMD/M06/SDD,6-31G(d)) = -2402.87743940

C	-0.33476400	1.91213700	-0.86627900
C	0.48736300	0.72187300	-0.47024900
C	-2.30797500	-1.71514000	-4.17827500
C	-1.37052000	-2.51285300	-3.34621100
C	-0.74907800	-2.05875600	-2.17616900
C	-1.19889100	-0.95342600	-1.40009200
C	-0.44998900	-0.50054800	-0.18421900
O	-0.37919700	2.26837300	-2.09960800
H	1.01461600	0.93590900	0.46742100
C	1.49949700	0.30176700	-1.51304900
H	-3.21894700	-2.29389100	-4.38410900
H	-2.59113500	-0.74962000	-3.75020600
H	-1.16143700	-0.21104700	0.59853500
H	0.16345700	-1.31495100	0.21915900
O	2.40574400	-0.49371300	-1.01711600
O	1.42614500	0.62023400	-2.69704500
C	3.37987900	-1.06513800	-1.94136000
C	4.20849300	-2.03812700	-1.15552300

H	3.96877800	-0.23815300	-2.35461200
H	2.82705900	-1.54291900	-2.75878400
H	4.95512200	-2.49399900	-1.81588700
H	3.58448400	-2.83743300	-0.73773700
H	4.73526600	-1.53711700	-0.33484800
C	-1.15063600	2.63118100	0.06049500
C	-2.06971500	3.58374600	-0.43424300
C	-1.07026700	2.39292700	1.44934000
C	-2.89297000	4.26461000	0.44043400
H	-2.13616700	3.76532400	-1.50402400
C	-1.89683900	3.08448500	2.31455900
H	-0.35698700	1.67967100	1.85570700
C	-2.80824800	4.01370200	1.81167700
H	-3.60838400	4.98974700	0.06071800
H	-1.83538100	2.90164900	3.38425500
H	-3.46174000	4.54951700	2.49747400
H	0.02583200	-2.69431300	-1.74023800
H	0.26666700	1.69699700	-2.64107600
Au	-2.70647300	-2.45173100	-0.97443600
Cl	-4.08945200	-0.75180500	0.20900000
Cl	-1.04034800	-3.73464000	0.85768700
Cl	-4.33146100	-4.25256900	-1.42296500
H	-1.82930800	-1.54072400	-5.15480300
C	-0.99510100	-3.84235600	-3.88429800
H	-0.44356900	-3.71066800	-4.82859300
H	-1.89750700	-4.41712700	-4.13623200
H	-0.38085100	-4.42135000	-3.18649000
H	-1.83076400	-0.19972500	-1.88260800

22

E (SMD/M06/SDD,6-31G(d)) = -2402.44779330

G (SMD/M06/SDD,6-31G(d)) = -2402.146869

E (SMD/M06/def2-TZVP//SMD/M06/SDD,6-31G(d)) = -2402.88573941

C	0.61330900	0.89041200	0.19149900
C	0.72670300	-0.58434800	-0.04453300
C	-0.70505800	-1.20360800	-0.26614400
C	-1.30551500	-0.78839500	-1.59259900
C	-0.91813400	-1.46763100	-2.83346500
C	-0.68723900	-2.78306700	-3.07855300
C	-0.24550400	-3.21137000	-4.44016100
O	0.88981600	1.70609100	-0.76035300
C	-0.83256500	-3.89782600	-2.09232000
H	1.12500500	-1.06225700	0.85838800
C	1.63575800	-0.93635200	-1.20100400
H	-1.32813000	-0.86920900	0.57014000
H	-0.58980800	-2.28577400	-0.16573600
H	-1.22112600	0.29787400	-1.72128600

H	-1.03673100	-4.84215500	-2.61398900
H	-1.63813000	-3.72849100	-1.36734100
H	0.10313000	-4.04535200	-1.52890500
H	-1.00960200	-3.85328600	-4.90547800
H	0.66552300	-3.82787700	-4.37446600
H	-0.04949600	-2.36500000	-5.10892600
O	2.03415300	-2.18018300	-1.15393900
O	1.94605800	-0.14478300	-2.08654700
C	2.86121100	-2.64498900	-2.26131800
C	3.16716300	-4.09337300	-2.01589600
H	3.76341200	-2.02299200	-2.28748300
H	2.29866600	-2.47816200	-3.18887600
H	3.80302700	-4.46893800	-2.82613900
H	2.25250000	-4.69927700	-1.99157000
H	3.70292400	-4.22960300	-1.06884900
C	0.14296400	1.44828400	1.42307300
C	-0.09717700	2.83820700	1.49364500
C	-0.08405000	0.64191400	2.55837800
C	-0.56091300	3.39897600	2.66751500
H	0.07359900	3.45966000	0.61824000
C	-0.54400300	1.21589600	3.72905600
H	0.10488800	-0.42910600	2.53260200
C	-0.78443400	2.58928800	3.78282200
H	-0.75373200	4.46761900	2.72027100
H	-0.71699600	0.59577000	4.60496700
H	-1.15023200	3.03489300	4.70588600
Au	-3.47006300	-0.86989300	-1.59913300
Cl	-3.52964000	-2.60068800	0.05004800
Cl	-5.96602400	-0.98198900	-1.82457500
Cl	-3.36069600	1.00808500	-3.09225600
H	-0.83683900	-0.79666400	-3.69264200
H	1.29678600	1.18833700	-1.53868300

$TS_{22\text{-}23}$

E (SMD/M06/SDD,6-31G(d)) = -2402.43465420

G (SMD/M06/SDD,6-31G(d)) = -2402.133055

E (SMD/M06/def2-TZVP//SMD/M06/SDD,6-31G(d)) = -2402.87298933

C	0.17888700	1.49411100	-1.23110900
C	1.14693000	0.28468100	-1.41392300
C	0.52534700	-1.00815800	-0.82081900
C	-0.95602900	-0.82842200	-1.04983100
C	-1.28791300	0.51240400	-0.49515800
C	-2.38702100	1.28898100	-0.90626200
C	-2.78191700	2.45994500	-0.11647200
O	-0.19338500	2.12619600	-2.36011100
C	-3.09395000	1.05421600	-2.17057100
H	2.08838400	0.51503700	-0.90178100

C	1.46623700	-0.01789900	-2.85930200
H	0.93081600	-1.90922600	-1.29698200
H	0.75706500	-1.06154500	0.24977800
H	-1.20727000	-0.92170800	-2.11252600
H	-3.52452500	1.97792400	-2.57475400
H	-2.49081000	0.54061500	-2.92741400
H	-3.94103500	0.38185200	-1.93932500
H	-2.38626700	3.35129600	-0.64201400
H	-3.87361000	2.58452100	-0.12278900
H	-2.39652500	2.45359400	0.90725300
O	2.64979100	-0.58125200	-2.97627100
O	0.70159000	0.16390500	-3.79733400
C	3.04151000	-1.01832800	-4.30389800
C	4.41431300	-1.61522200	-4.18507800
H	2.29767500	-1.74212000	-4.65827700
H	3.01822800	-0.14731700	-4.96979000
H	4.75084600	-1.95774700	-5.17056000
H	5.13447900	-0.87613600	-3.81376200
H	4.41246500	-2.47528000	-3.50467200
C	0.53965200	2.47113300	-0.16158900
C	1.06421900	2.04353000	1.06564900
C	0.34442300	3.83971100	-0.37739100
C	1.38065100	2.96424900	2.05338400
H	1.21903000	0.98221100	1.26197600
C	0.66257500	4.75992800	0.61696300
H	-0.04256300	4.18407200	-1.33334300
C	1.17732500	4.32644800	1.83334900
H	1.78701200	2.61717200	3.00136800
H	0.50869400	5.82186000	0.43427100
H	1.42379200	5.04657500	2.61147600
Au	-2.09800700	-2.36625900	-0.16131200
H	-1.05601900	0.61135600	0.56757700
H	-0.13818900	1.49531900	-3.11974300
Cl	-1.35912100	-1.67026400	1.98362200
Cl	-3.47113400	-4.15642500	0.84717100
Cl	-2.74235300	-3.01746100	-2.34223800

23

E (SMD/M06/SDD,6-31G(d)) = -2402.43485204

G (SMD/M06/SDD,6-31G(d)) = -2402.135109

E (SMD/M06/def2-TZVP//SMD/M06/SDD,6-31G(d)) = -2402.87355477

C	0.07719100	1.40204100	-1.17967200
C	1.11309300	0.23595100	-1.38621300
C	0.48932300	-1.06762900	-0.82235800
C	-0.98713300	-0.85402600	-1.05830400
C	-1.23946500	0.52885800	-0.52520300
C	-2.42767000	1.25697700	-0.87735700

C	-2.82224000	2.41161300	-0.08049200
O	-0.28981400	2.05623800	-2.33403000
C	-3.21020200	0.92546200	-2.06063000
H	2.04371300	0.47551000	-0.86048900
C	1.44873900	-0.04075000	-2.83114200
H	0.87939100	-1.96769000	-1.31420900
H	0.71656600	-1.14287400	0.24908400
H	-1.22577400	-0.93717000	-2.12489300
H	-3.81436200	1.76675400	-2.41621000
H	-2.61824400	0.47601700	-2.86637500
H	-3.91581600	0.13582500	-1.73017000
H	-2.42497100	3.29127100	-0.62978200
H	-3.91167700	2.54737600	-0.07869500
H	-2.40711900	2.41976900	0.93112300
O	2.66815800	-0.52857600	-2.95160400
O	0.67385000	0.08853800	-3.76847800
C	3.07816500	-0.94341500	-4.27885900
C	4.47944700	-1.47181300	-4.16442600
H	2.37156400	-1.70315800	-4.63454700
H	3.01048800	-0.07470100	-4.94505000
H	4.83098200	-1.79601400	-5.15091400
H	5.16304900	-0.69886800	-3.79285700
H	4.52145700	-2.33189000	-3.48525200
C	0.52613000	2.41507100	-0.15465600
C	1.00426300	2.00321600	1.09557400
C	0.47347100	3.78032300	-0.44193100
C	1.41879700	2.93676200	2.03525300
H	1.05350400	0.94164800	1.34423500
C	0.88699700	4.71574900	0.50435000
H	0.11845100	4.10967800	-1.41522100
C	1.35884700	4.29908300	1.74319200
H	1.78987500	2.59918400	3.00130000
H	0.84099000	5.77685200	0.26483500
H	1.68236100	5.03049400	2.48175300
Au	-2.16227800	-2.37120500	-0.19364600
H	-1.06628100	0.57924000	0.55403800
H	-0.22932100	1.42682300	-3.08698600
Cl	-1.76403900	-1.47583400	1.96445800
Cl	-3.56135400	-4.16156900	0.76975700
Cl	-2.44681100	-3.23007000	-2.37746600

$TS_{23\text{-}24}$
E (SMD/M06/SDD,6-31G(d)) = -2402.42872943
G (SMD/M06/SDD,6-31G(d)) = -2402.125243
E (SMD/M06/def2-TZVP//SMD/M06/SDD,6-31G(d)) = -2402.86465316

C	-0.17756000	0.96558200	-0.42686600
C	0.89317500	0.12502900	-1.08423900

C	0.38081100	-1.30627500	-0.73841600
C	-1.12547900	-1.18969200	-0.88363700
C	-1.49619600	0.21426100	-0.39452000
C	-2.34409800	1.12331300	-1.32016300
C	-3.23572800	2.06229100	-0.53888400
O	-1.29285200	1.95933700	-1.91451600
C	-3.14069600	0.42708200	-2.39838200
H	1.86094700	0.31357400	-0.60975500
C	1.10468500	0.21025600	-2.58487300
H	0.82359900	-2.06831100	-1.39106400
H	0.68022500	-1.52446200	0.29405000
H	-1.42529800	-1.35666200	-1.92093700
H	-3.69010000	1.17812600	-2.98097300
H	-2.51511200	-0.14566500	-3.09376600
H	-3.87109800	-0.25991700	-1.95041700
H	-3.71525600	2.77141400	-1.22517100
H	-4.01737200	1.49177000	-0.02061100
H	-2.67460600	2.64056900	0.20445300
O	2.36105600	-0.06901500	-2.86685200
O	0.25028500	0.42520700	-3.42651800
C	2.71740300	-0.13769300	-4.27301500
C	4.18520800	-0.44692400	-4.34091600
H	2.10118300	-0.91377500	-4.74261600
H	2.46810800	0.82519400	-4.73458900
H	4.49715800	-0.50943300	-5.38992300
H	4.77655800	0.33735800	-3.85309000
H	4.40886700	-1.40596900	-3.85848800
C	0.19181300	2.03834500	0.44223500
C	-0.55128300	2.33620700	1.60328600
C	1.32570800	2.82040500	0.12674600
C	-0.16468000	3.38243800	2.42421300
H	-1.40151500	1.71848000	1.88464400
C	1.68120900	3.88498800	0.93135200
H	1.89658500	2.60658400	-0.77685800
C	0.94023800	4.16108000	2.08366400
H	-0.72365300	3.59330800	3.33270900
H	2.53944500	4.50075000	0.67317600
H	1.23381500	4.98913100	2.72610500
Au	-2.12182400	-2.74079800	0.12994800
H	-1.91569100	0.18466200	0.61855500
H	-0.95639100	1.50007200	-2.71874600
Cl	-1.30915000	-1.88904200	2.18807600
Cl	-3.27328000	-4.58941900	1.29330800
Cl	-2.86211400	-3.52573800	-1.97001600

24

E (SMD/M06/SDD,6-31G(d)) = -2402.44665965

G (SMD/M06/SDD,6-31G(d)) = -2402.142889
E (SMD/M06/def2-TZVP//SMD/M06/SDD,6-31G(d)) = -2402.88331421

C	-0.04302700	0.19715400	-0.30264400
C	0.79297100	-0.92124400	-0.83728800
C	-0.15426500	-2.12749600	-0.93292800
C	-1.55837900	-1.57523800	-0.70777400
C	-1.45379800	-0.05210100	-0.67443200
C	-1.80890500	0.57591500	-2.11600800
C	-1.45209400	2.04743100	-2.18865700
O	-1.05732400	-0.06512400	-3.12311600
C	-3.30149800	0.39461200	-2.34260900
H	1.65009900	-1.12556800	-0.18045800
C	1.41711200	-0.58927900	-2.20684200
H	-0.07812100	-2.59080000	-1.92474300
H	0.12291100	-2.89879600	-0.20678200
H	-2.28231800	-1.94985200	-1.43392900
H	-3.55898100	0.80866900	-3.32621600
H	-3.60952200	-0.65892600	-2.32783800
H	-3.88153500	0.92768600	-1.57862300
H	-1.79689300	2.43979100	-3.15381000
H	-1.94501700	2.61859500	-1.39243200
H	-0.36886200	2.20860400	-2.12649100
O	1.69676600	0.70245700	-2.31021800
O	1.68045300	-1.43012200	-3.02846100
C	2.24598200	1.15254500	-3.56783000
C	2.54612500	2.61696300	-3.41344000
H	3.14332500	0.56290400	-3.79103700
H	1.49876100	0.95296000	-4.34773700
H	2.98569000	3.00673900	-4.33898700
H	1.63329900	3.18812200	-3.20107500
H	3.25917200	2.78507000	-2.59613200
C	0.44494400	1.31767000	0.40675100
C	-0.44870600	2.26364700	0.97436400
C	1.84026200	1.51463200	0.57910600
C	0.03295500	3.36518900	1.65055400
H	-1.52311400	2.11305900	0.89976900
C	2.31294500	2.62355800	1.24869200
H	2.54709700	0.79719100	0.17153800
C	1.41151200	3.54853700	1.78298400
H	-0.65804600	4.08117600	2.08823100
H	3.38304800	2.77246000	1.36969000
H	1.78845300	4.41618000	2.32146100
Au	-2.42700500	-2.24664200	1.10383500
H	-2.16707400	0.42356000	0.00923000
H	-1.49521500	-0.89784900	-3.36865000
Cl	-0.52679100	-1.39727000	2.24714200
Cl	-3.45778800	-3.01178700	3.22992500

Cl	-4.28255700	-2.99696400	-0.15838600

TS_{24-25}

E (SMD/M06/SDD,6-31G(d)) = -2402.43762241
G (SMD/M06/SDD,6-31G(d)) = -2402.135002
E (SMD/M06/def2-TZVP//SMD/M06/SDD,6-31G(d)) = -2402.87413925

C	-0.39028800	0.86435700	-1.16617900
C	0.61543700	-0.18317800	-1.56151400
C	-0.01716800	-1.53452300	-1.13702200
C	-1.39380900	-1.18244400	-0.59078600
C	-1.40067800	0.28891200	-0.37565500
C	-0.81858500	0.73405300	1.52716900
C	-0.10961400	-0.47721400	2.05525600
C	-2.17274500	0.98767200	2.11219000
H	0.78171300	-0.16042200	-2.64628500
C	1.94887500	0.04272800	-0.87962100
H	-0.04398700	-2.23862500	-1.97504600
H	0.57061600	-2.02237400	-0.34475300
H	-1.71292400	-1.79043000	0.25746900
H	-2.03734800	1.15073300	3.19211100
H	-2.63438800	1.88572000	1.68632300
H	-2.83517000	0.12547600	1.97607500
H	0.08744200	-0.26209200	3.11693200
H	-0.72748000	-1.37812700	2.01476400
H	0.85723500	-0.65868000	1.57571600
O	2.89862700	-0.67450200	-1.44559400
O	2.12643500	0.76688100	0.08810600
C	4.22079400	-0.60464400	-0.85252900
C	5.11701400	-1.49580100	-1.66375500
H	4.54594400	0.44256100	-0.86642400
H	4.14402100	-0.92588000	0.19341000
H	6.13139200	-1.47125900	-1.24880100
H	4.76297700	-2.53353500	-1.64497600
H	5.16458400	-1.16155100	-2.70714200
C	-0.25296700	2.24652100	-1.49348300
C	0.82371600	2.69525200	-2.29139800
C	-1.18997100	3.19535200	-1.02506700
C	0.95512300	4.03472600	-2.60540500
H	1.55268300	1.98628800	-2.67993600
C	-1.04773100	4.53535100	-1.33515300
H	-2.03261700	2.87483700	-0.41647600
C	0.02210100	4.95580800	-2.12548700
H	1.78287400	4.36870800	-3.22669700
H	-1.77275200	5.25769600	-0.96742400
H	0.12728700	6.01006100	-2.37519200
Au	-2.99709300	-1.64493600	-1.94309200
H	-2.35852600	0.80010900	-0.28030400

Cl	-1.88659900	-0.46609100	-3.68554300
Cl	-4.88050100	-2.23457100	-3.47336900
Cl	-4.06777700	-2.72211500	-0.11705800
O	-0.09051600	1.85205000	1.47308200
H	0.81904300	1.62974300	1.16156300

25

E (SMD/M06/SDD,6-31G(d)) = -2208.99241308

G (SMD/M06/SDD,6-31G(d)) = -2208.786823

E (SMD/M06/def2-TZVP//SMD/M06/SDD,6-31G(d)) = -2209.35396206

C	-0.50459200	0.70941300	-1.12362800
C	0.45894800	-0.43568700	-1.35166800
C	-0.23726000	-1.62702200	-0.66708300
C	-1.68148700	-1.20648900	-0.52633700
C	-1.69026400	0.23706000	-0.65503200
H	0.58024200	-0.62547000	-2.42841400
C	1.81267100	-0.18556000	-0.72754000
H	-0.08077700	-2.57455000	-1.19049100
H	0.15698100	-1.74054200	0.35590400
H	-2.24449300	-1.66432100	0.28991600
O	2.80976800	-0.48685400	-1.56350900
O	1.97566100	0.21838100	0.40309200
C	4.14268400	-0.28690200	-1.05478000
C	5.10296300	-0.70092000	-2.13486500
H	4.25455100	0.77097300	-0.78169100
H	4.26352000	-0.88279200	-0.14103300
H	6.13441400	-0.56387600	-1.78887400
H	4.96628700	-1.75713800	-2.39772500
H	4.96124600	-0.09783700	-3.04022000
C	-0.17317100	2.09828400	-1.41716200
C	0.96144900	2.41924200	-2.17968100
C	-0.98214700	3.14977400	-0.95340700
C	1.26898100	3.74142800	-2.47734000
H	1.60228700	1.62696200	-2.56594300
C	-0.67083200	4.46847200	-1.24816300
H	-1.85559600	2.92924000	-0.34149200
C	0.45563300	4.77083300	-2.01279200
H	2.14889800	3.96715600	-3.07748800
H	-1.30859600	5.26845600	-0.87564600
H	0.69850900	5.80691100	-2.24283300
Au	-3.05258200	-1.81226500	-2.13441000
H	-2.57051900	0.84160700	-0.44589500
Cl	-1.27836300	-2.35789200	-3.64566900
Cl	-4.78556800	-2.29485800	-3.94281800
Cl	-4.79262200	-1.40395200	-0.52346400

26

E (SMD/M06/SDD,6-31G(d)) = -193.440144261
G (SMD/M06/SDD,6-31G(d)) = -193.371864
E (SMD/M06/def2-TZVP//SMD/M06/SDD,6-31G(d)) = -193.521800782

C	-0.95342800	0.74743200	1.71958500
C	-0.30303200	-0.48768800	2.14951500
C	-2.34839600	1.04361300	2.02924200
H	-2.59112100	0.70020300	3.04187000
H	-2.58674400	2.10037500	1.88579800
H	-2.96389400	0.43777700	1.34362900
H	-0.05456000	-0.35636400	3.21644400
H	-1.00951000	-1.32459900	2.10116100
H	0.61611700	-0.70795300	1.59656700
O	-0.32319700	1.63957900	1.05593200
H	0.60930400	1.38118100	0.85865200

27

E (SMD/M06/SDD,6-31G(d)) = -2402.46135889
G (SMD/M06/SDD,6-31G(d)) = -2402.166721
E (SMD/M06/def2-TZVP//SMD/M06/SDD,6-31G(d)) = -2402.90110629

C	-0.40125200	0.53781800	-0.90252500
C	0.49047200	-0.60306700	-1.35294900
C	-0.22600500	-1.84871500	-0.79591100
C	-1.65290900	-1.39768200	-0.56493400
C	-1.59350000	0.05243900	-0.47582500
H	0.54585700	-0.64871700	-2.45061000
C	1.87983800	-0.47798200	-0.79726000
H	-0.11405800	-2.72722500	-1.43709600
H	0.19023200	-2.09780400	0.19348100
H	-2.20192500	-1.93778500	0.21044900
O	2.82585700	-0.65519100	-1.69147200
O	2.08545800	-0.23378000	0.38960600
C	4.20036200	-0.50556600	-1.26181100
C	5.07387200	-0.79005600	-2.44947300
H	4.32595800	0.52402500	-0.89641400
H	4.38493200	-1.20381000	-0.43542200
H	6.12660400	-0.68091800	-2.16468100
H	4.91920500	-1.81322900	-2.81144200
H	4.86314500	-0.09252400	-3.26863000
C	0.01559000	1.93504000	-0.95193100
C	1.09322900	2.33601600	-1.75856300
C	-0.63325400	2.90850900	-0.17446600
C	1.50015800	3.66566400	-1.79404500
H	1.59964500	1.60980000	-2.39466300
C	-0.22216700	4.23392500	-0.20830400
H	-1.45637200	2.61663100	0.47665900
C	0.84673600	4.61888800	-1.01709200
H	2.32752900	3.95827300	-2.43844600

H	-0.73691000	4.97257500	0.40394700
H	1.16664500	5.65909000	-1.04270500
Au	-3.07634000	-1.74420000	-2.18090400
H	-2.43550400	0.65980600	-0.14987400
Cl	-1.36996300	-2.19225200	-3.79122200
Cl	-4.85393000	-2.05344600	-3.95442900
Cl	-4.74950500	-1.35143900	-0.50646600
C	3.76442400	1.79807800	2.24816800
C	2.65845000	2.56028400	1.64771800
C	4.68382600	2.40730400	3.20705900
H	4.09534800	2.80743500	4.04594700
H	5.43991700	1.70403100	3.56284500
H	5.15294200	3.28200500	2.73288700
H	1.70381700	2.08145500	1.91190400
H	2.65679500	3.59769000	1.98977900
H	2.73000500	2.52900700	0.54883000
O	3.95480300	0.57766400	1.95489900
H	3.27128800	0.21076100	1.28436200

TS_{27-28}

E (SMD/M06/SDD,6-31G(d)) = -2402.42406517

G (SMD/M06/SDD,6-31G(d)) = -2402.135893

E (SMD/M06/def2-TZVP//SMD/M06/SDD,6-31G(d)) = -2402.86498095

C	-0.40494200	0.43718500	-1.95830700
C	0.75380800	-0.55739300	-2.13592000
C	0.04374800	-1.92675200	-2.14706800
C	-1.26867700	-1.69307700	-1.40113200
C	-1.46647500	-0.25692700	-1.37411500
H	1.32772000	-0.38106400	-3.05175700
C	1.65891700	-0.44327600	-0.92599500
H	-0.14877800	-2.21800500	-3.18920800
H	0.63851000	-2.72699900	-1.69371300
H	-1.32751500	-2.09801100	-0.38045300
O	2.93261000	-0.29707400	-1.27204500
O	1.25303900	-0.49711500	0.21499600
C	3.88316300	-0.21326400	-0.18688800
C	5.24637600	-0.05353700	-0.79862400
H	3.60823100	0.63778600	0.44877500
H	3.80113800	-1.12580900	0.41687900
H	6.00189500	0.01569800	-0.00710500
H	5.49472500	-0.91110200	-1.43589700
H	5.30033100	0.85851400	-1.40565800
C	-0.13151500	1.88885200	-1.77234500
C	0.83229900	2.50322900	-2.58033000
C	-0.83556800	2.66216800	-0.84356900
C	1.08306000	3.86606500	-2.46446300
H	1.38311400	1.91354800	-3.31360700

C	-0.58421800	4.02467600	-0.73199500
H	-1.57451800	2.19643600	-0.19279200
C	0.37289200	4.63057300	-1.54280600
H	1.83495600	4.33211700	-3.09887700
H	-1.13334600	4.61460700	-0.00020400
H	0.56908900	5.69747300	-1.45155800
Au	-2.86586500	-2.78082000	-2.28622400
H	-2.35004700	0.21428000	-0.94438900
Cl	-3.80340200	-0.79950300	-3.19473400
Cl	-4.62804400	-4.13136900	-3.32095200
Cl	-1.87315200	-4.69525400	-1.31594900
C	-1.78608200	1.07972500	-5.12320700
C	-2.47044600	2.19682200	-4.41891300
C	-2.07859500	0.89604100	-6.56728900
H	-2.15205900	1.86236100	-7.08071700
H	-1.33728700	0.25072200	-7.04669300
H	-3.06803900	0.41851500	-6.64198600
H	-1.94112900	3.12619700	-4.68226600
H	-3.50011800	2.30529000	-4.78053000
H	-2.46207200	2.08889000	-3.32976300
O	-0.99047000	0.31382000	-4.56228700
H	-0.90496300	0.37523700	-3.12391000

28

E (SMD/M06/SDD,6-31G(d)) = -2209.40669716

G (SMD/M06/SDD,6-31G(d)) = -2209.183752

E (SMD/M06/def2-TZVP//SMD/M06/SDD,6-31G(d)) = -2209.76603601

C	-0.39866800	0.42548800	-2.21442800
C	0.78286400	-0.44858100	-2.68505000
C	0.15914100	-1.87473700	-2.60777900
C	-0.69752700	-1.81586700	-1.33094000
C	-0.55403400	-0.36390200	-0.91346800
H	1.29651600	-0.21214300	-3.61787500
C	1.61324900	-0.35922100	-1.46262400
H	-0.43716900	-2.01348200	-3.51797900
H	0.91251900	-2.66959400	-2.57881700
H	-0.35299500	-2.48364900	-0.53422200
O	2.87948600	-0.38164700	-1.41903300
O	0.88480300	-0.30951100	-0.40264300
C	3.56357300	-0.31577300	-0.10379700
C	5.03294400	-0.30535100	-0.38729000
H	3.20875500	0.59678700	0.38735300
H	3.23641500	-1.19238400	0.46470400
H	5.57322900	-0.25944800	0.56516000
H	5.34148100	-1.21568900	-0.91366900
H	5.31497500	0.56886300	-0.98496500
C	-0.15148500	1.90083200	-2.03910400

C	1.00837300	2.52890200	-2.49557300
C	-1.12811500	2.66972900	-1.39700000
C	1.19343400	3.89752800	-2.30531200
H	1.77782600	1.96219500	-3.02060000
C	-0.94469600	4.03353600	-1.20850400
H	-2.04287700	2.18907600	-1.04704600
C	0.22019900	4.65173100	-1.66059100
H	2.10361300	4.37219100	-2.66812700
H	-1.71477800	4.61758500	-0.70727300
H	0.36479800	5.72060100	-1.51266400
Au	-2.64948000	-2.52032000	-1.66445800
H	-1.15942600	0.02802200	-0.09796400
Cl	-3.62279400	-0.40450100	-1.26226700
Cl	-4.86551400	-3.47447700	-2.10827000
Cl	-1.61338000	-4.62158500	-1.99876900
H	-1.27430400	0.24699400	-2.85293700

29

E (SMD/M06/SDD,6-31G(d)) = -693.081070091

G (SMD/M06/SDD,6-31G(d)) = -692.853079

E (SMD/M06/def2-TZVP//SMD/M06/SDD,6-31G(d)) = -693.347414360

C	-0.48492129	0.34884165	2.40429561
C	0.75927762	-0.52646764	2.49632984
C	0.13386612	-1.91636451	2.45450091
C	-0.89207515	-1.71580115	1.31423619
C	-1.25528901	-0.38943217	1.28575894
H	1.36548349	-0.34178760	3.35848280
C	1.63228522	-0.31532450	1.24537968
H	-0.37458149	-2.14792828	3.36705734
H	0.85342706	-2.68660612	2.27049025
H	-1.25572686	-2.47867022	0.65796778
O	2.86924330	-0.54104343	1.29593869
O	1.09084952	0.09650535	0.13604418
C	3.67989371	-0.34497903	0.13434294
C	5.10406361	0.05385668	0.56360995
H	3.25806137	0.43234790	-0.46791972
H	3.71877598	-1.25271356	-0.43081738
H	5.71063419	0.20056221	-0.30555610
H	5.52589595	-0.72347026	1.16587261
H	5.06518134	0.96159121	1.12877028
C	-0.15584703	1.81949139	2.08727911
C	1.02575064	2.39394567	2.57487648
C	-1.03798713	2.58332839	1.31119673
C	1.32520893	3.73223651	2.28639025
H	1.69928294	1.81073991	3.16743473
C	-0.73852810	3.92161879	1.02270922
H	-1.94016478	2.14472076	0.93890809

C	0.44307050	4.49607252	1.51030500
H	2.22738488	4.17084515	2.65868182
H	-1.41206215	4.50482560	0.43015399
H	0.67171451	5.51788671	1.29003770
H	-1.95022108	0.05552888	0.60459885
H	-1.04982780	0.32472419	3.31270024

TS_{27-30}

E (SMD/M06/SDD,6-31G(d)) = -2402.44101162
G (SMD/M06/SDD,6-31G(d)) = -2402.146253
E (SMD/M06/def2-TZVP//SMD/M06/SDD,6-31G(d)) = -2402.87376759

C	-0.75075800	0.53640300	-1.38137600
C	0.28028000	-0.59097800	-1.32057000
C	-0.50566700	-1.81127500	-0.79901200
C	-1.90850100	-1.33071600	-0.72249900
C	-2.04989900	-0.04266900	-1.19084900
H	1.12325200	-0.33557200	-0.66695400
C	0.78452600	-0.82753500	-2.72484400
H	-0.44709500	-2.67447200	-1.47967500
H	-0.13137000	-2.16108800	0.17248100
O	2.07924900	-0.67806600	-2.84582200
O	0.01975000	-1.11645700	-3.64064400
C	2.66005800	-0.78737300	-4.17129200
C	4.14592600	-0.63984600	-4.01654000
H	2.37914100	-1.76047800	-4.59379200
H	2.23039700	0.01560500	-4.78751100
H	4.62464700	-0.71049500	-4.99984000
H	4.39954200	0.33216300	-3.57730100
H	4.55305000	-1.43176200	-3.37718300
C	-0.45643100	1.79705400	-2.04097100
C	0.87206700	2.24998500	-2.14130400
C	-1.48158500	2.56319600	-2.62355700
C	1.16451900	3.42471800	-2.81945400
H	1.67676100	1.69512100	-1.66207700
C	-1.18187600	3.73318200	-3.30487100
H	-2.51553400	2.23281700	-2.55428200
C	0.13968700	4.16640500	-3.40518400
H	2.19482900	3.76902100	-2.88193500
H	-1.98357900	4.31409700	-3.75662700
H	0.37060300	5.08891700	-3.93499700
Au	-1.28724900	0.79097800	0.90473200
Cl	-2.61596500	3.03313500	0.55213500
Cl	-2.32237900	-0.07286200	3.02857900
Cl	1.15843200	0.98107600	1.59133700
H	-2.99682800	0.47159800	-1.32806900
H	-2.73684900	-1.95647600	-0.39740100
C	-0.66804100	-0.05381800	-6.61213500

C	-1.01328800	1.00413600	-5.64986000
C	-0.94592100	0.08498400	-8.03935600
H	-0.39045800	0.95810500	-8.41392100
H	-0.68404200	-0.81203000	-8.60470900
H	-2.00889100	0.33682600	-8.16690200
H	-0.16918600	1.20837400	-4.97604700
H	-1.32980500	1.91896700	-6.15631000
H	-1.84068800	0.63598900	-5.02180000
O	-0.12618800	-1.13528400	-6.22493000
H	0.02835800	-1.16415200	-5.21719800

30

E (SMD/M06/SDD,6-31G(d)) = -2402.88914118

G (SMD/M06/SDD,6-31G(d)) = -2402.153028

E (SMD/M06/def2-TZVP//SMD/M06/SDD,6-31G(d)) = -2402.90138127

C	-0.86481100	0.41746300	-1.10767000
C	0.19808800	-0.68783000	-1.27006600
C	-0.53156400	-1.98981200	-0.88753800
C	-1.96998500	-1.62638600	-1.03220400
C	-2.14463300	-0.29891800	-1.16372900
H	1.09184700	-0.52134800	-0.66580900
C	0.63384100	-0.77186400	-2.71232800
H	-0.23653400	-2.84623400	-1.50991600
H	-0.29594200	-2.26330400	0.15368600
O	1.94233100	-0.74638800	-2.83886500
O	-0.15006600	-0.88309900	-3.65194600
C	2.51150900	-0.83463100	-4.16594600
C	4.00510100	-0.85335200	-4.00801000
H	2.13794600	-1.74835000	-4.64658500
H	2.17492200	0.04095000	-4.73879000
H	4.47808500	-0.91424700	-4.99483200
H	4.35762400	0.05803700	-3.51104600
H	4.32463600	-1.72061100	-3.41844800
C	-0.61912800	1.67869500	-1.84714000
C	0.67022900	2.23713400	-1.86547600
C	-1.63276000	2.31411100	-2.57863500
C	0.93467200	3.39488700	-2.58669000
H	1.46839800	1.76953800	-1.28822900
C	-1.36641300	3.47456800	-3.29597700
H	-2.63615300	1.89371200	-2.59414200
C	-0.08458700	4.02072600	-3.30274400
H	1.93888700	3.81526000	-2.58100000
H	-2.16706500	3.95335500	-3.85811000
H	0.11961400	4.93204800	-3.86232500
Au	-0.93042100	1.15135600	1.04524300
Cl	-2.95913000	2.35007800	0.51124900
Cl	-1.16389600	1.94584400	3.45213100

Cl	1.13974700	0.10216400	1.65871200
H	-3.10596200	0.20155600	-1.22765000
H	-2.77305800	-2.35907400	-0.99923100
C	-0.52170800	0.15023100	-6.71569700
C	-0.82921400	1.29893900	-5.85137800
C	-0.66479700	0.22534400	-8.16864400
H	0.05554300	0.96960200	-8.54151000
H	-0.50129400	-0.73967700	-8.65355300
H	-1.65811500	0.62911200	-8.40982800
H	-0.07815400	1.41996800	-5.05957500
H	-0.93238100	2.22028800	-6.43010200
H	-1.78869800	1.09037100	-5.35065700
O	-0.13754900	-0.95338500	-6.22172200
H	-0.06200100	-0.93737800	-5.19761300

$TS_{30\text{-}31}$
E (SMD/M06/SDD,6-31G(d)) = -2402.44101162
G (SMD/M06/SDD,6-31G(d)) = -2402.146253
E (SMD/M06/def2-TZVP//SMD/M06/SDD,6-31G(d)) = -2402.87376759

C	-0.19693100	0.42709100	-1.41991600
C	0.72795500	-0.72985400	-1.81164400
C	0.21125700	-1.90502400	-0.95437000
C	-1.27031700	-1.59642300	-0.85303600
C	-1.43440100	-0.23029900	-1.02589800
H	1.78761800	-0.52871500	-1.62663600
C	0.57441600	-1.08000000	-3.28243000
H	0.43398100	-2.88424000	-1.39444700
H	0.67980300	-1.87004900	0.04271600
O	1.55765000	-1.88919400	-3.66426500
O	-0.32488100	-0.71583000	-4.01230500
C	1.49578500	-2.37672700	-5.02134800
C	2.68516700	-3.27086700	-5.23067800
H	0.54503000	-2.91097600	-5.15383500
H	1.49683700	-1.51545700	-5.70136800
H	2.67796400	-3.66362200	-6.25429700
H	3.62150600	-2.71957700	-5.08105600
H	2.66675200	-4.11966000	-4.53623000
C	-0.20682500	1.70369600	-2.16519800
C	0.88283400	2.06697100	-2.97201500
C	-1.29218100	2.59045700	-2.06731100
C	0.88049000	3.26849400	-3.66638800
H	1.74279600	1.40414000	-3.05499300
C	-1.29006300	3.79274000	-2.76139000
H	-2.14581100	2.34795100	-1.43560100
C	-0.20465400	4.13675100	-3.56405300
H	1.73273800	3.52967800	-4.29107000
H	-2.14090600	4.46581400	-2.67291600

H	-0.20344300	5.07982900	-4.10754400
Au	0.44962800	1.13246400	0.65476100
H	-2.39075100	0.27842400	-0.94555400
Cl	-1.76508200	1.03620300	1.56575600
Cl	1.19813300	2.10481800	2.78343700
Cl	2.70619700	1.21158800	-0.09616400
C	-2.70047000	-2.29592700	-3.94107400
C	-3.47033000	-1.04802100	-3.70662900
C	-2.72447900	-2.90832500	-5.28697300
H	-2.35736100	-2.16202200	-6.00763700
H	-2.11017800	-3.81144800	-5.33213500
H	-3.76018500	-3.13353300	-5.57392300
H	-2.77358400	-0.20364200	-3.82452300
H	-4.27305200	-0.92849100	-4.44052000
H	-3.87930800	-1.01694400	-2.69066500
O	-2.01182800	-2.82383900	-3.04841100
H	-1.78635800	-2.07639100	-1.99007600
H	-1.93954100	-2.15078900	-0.18710200

31

E (SMD/M06/SDD,6-31G(d)) = -2402.42254813

G (SMD/M06/SDD,6-31G(d)) = -2402.129995

E (SMD/M06/def2-TZVP//SMD/M06/SDD,6-31G(d)) = -2402.86147144

C	-0.09319700	0.43862800	-1.66889600
C	0.85642100	-0.69125000	-1.96538700
C	0.52624700	-1.76879300	-0.90952700
C	-0.93793600	-1.50666600	-0.56674200
C	-1.08218600	-0.00941200	-0.73084000
H	1.90965100	-0.38656300	-1.94506100
C	0.51561500	-1.24326500	-3.34167500
H	0.70902800	-2.78161800	-1.28559200
H	1.16729400	-1.61586900	-0.03240800
O	1.55242900	-1.89310100	-3.85317700
O	-0.57224300	-1.14275400	-3.86622100
C	1.33103300	-2.55106900	-5.12262800
C	2.61914200	-3.22272000	-5.50594000
H	0.50669300	-3.26524300	-5.00254500
H	1.02015400	-1.79466000	-5.85389400
H	2.49598600	-3.73589000	-6.46691200
H	3.42945700	-2.49071300	-5.60884400
H	2.91424300	-3.96572100	-4.75505000
C	-0.05958500	1.70413300	-2.31753900
C	0.99707400	2.02221200	-3.20176900
C	-1.07948200	2.66079400	-2.09357500
C	1.02703500	3.24660800	-3.84184800
H	1.79951700	1.30781400	-3.37657000
C	-1.04209300	3.88060800	-2.73734700

H	-1.89935200	2.43780100	-1.41312600
C	0.00993500	4.17411000	-3.61021700
H	1.84256600	3.48772600	-4.51913400
H	-1.82869200	4.61131900	-2.56588000
H	0.03688900	5.13875000	-4.11346800
Au	-0.45175200	0.98119500	1.13083600
H	-2.08442000	0.41355700	-0.81572100
Cl	-2.73361800	0.82092200	1.72586600
Cl	0.03170300	2.01318900	3.25723000
Cl	1.85132900	1.14830900	0.56038500
H	-1.22874200	-1.86950800	0.42572900
H	-1.60639100	-1.99267100	-1.29562000

acetone

E (SMD/M06/SDD,6-31G(d)) = -193.026272542

G (SMD/M06/SDD,6-31G(d)) = -192.969708

E (SMD/M06/def2-TZVP//SMD/M06/SDD,6-31G(d)) = -193.108777298

C	-2.75519800	-2.36374400	-3.88830300
C	-3.46434500	-1.05199400	-3.69947100
C	-2.66539800	-2.85921800	-5.30449300
H	-2.23855600	-2.08660600	-5.95839100
H	-2.05843000	-3.76852600	-5.35748800
H	-3.67364800	-3.07208900	-5.68707200
H	-2.90206700	-0.25094100	-4.19979100
H	-4.45740200	-1.07810900	-4.16835600
H	-3.56166400	-0.81570400	-2.63513600
O	-2.28406800	-2.98526100	-2.95398800
